# Bayesian subset selection and variable importance for interpretable prediction and classification

**Published:** 2022

**Authors:** Daniel R. Kowal

**Affiliations:** Department of Statistics, Rice University, Houston, TX 77005, USA

**Keywords:** education, linear regression, logistic regression, model selection, penalized regression

## Abstract

Subset selection is a valuable tool for interpretable learning, scientific discovery, and data compression. However, classical subset selection is often avoided due to selection instability, lack of regularization, and difficulties with post-selection inference. We address these challenges from a Bayesian perspective. Given any Bayesian predictive model ℳ, we extract a *family* of near-optimal subsets of variables for linear prediction or classification. This strategy deemphasizes the role of a single “best” subset and instead advances the broader perspective that often many subsets are highly competitive. The *acceptable family* of subsets offers a new pathway for model interpretation and is neatly summarized by key members such as the smallest acceptable subset, along with new (co-) variable importance metrics based on whether variables (co-) appear in all, some, or no acceptable subsets. More broadly, we apply Bayesian decision analysis to derive the optimal linear coefficients for *any* subset of variables. These coefficients inherit both regularization and predictive uncertainty quantification via ℳ. For both simulated and real data, the proposed approach exhibits better prediction, interval estimation, and variable selection than competing Bayesian and frequentist selection methods. These tools are applied to a large education dataset with highly correlated covariates. Our analysis provides unique insights into the combination of environmental, socioeconomic, and demographic factors that predict educational outcomes, and identifies over 200 distinct subsets of variables that offer near-optimal out-of-sample predictive accuracy.

## 1. Introduction

Subset and variable selection are essential components of regression analysis, prediction, and classification. By identifying subsets of important covariates, the analyst can acquire simpler and more interpretable summaries of the data, improved prediction or classification, reduced estimation variability among the selected covariates, lower storage requirements, and insights into the factors that determine predictive accuracy (Miller, 1984). Classical subset selection is expressed as the solution to the constrained least squares problem

(1) $$\underset{\beta }{\text{min}}∥y-X\beta {∥}_{2}^{2}\text{ subject to }∥\beta {∥}_{0}\leq k$$

where y is an n-dimensional response, X is an n×p matrix of covariates, and β is the p-dimensional vector of unknown coefficients. Traditionally, the goal is to determine (i) the best subset of each size k, (ii) an estimate of the accompanying nonzero linear coefficients, and (iii) the best subset of any size. Subset selection has garnered additional attention recently, in part due to the algorithmic advancements from Bertsimas et al. (2016) and the detailed comparisons of Hastie et al. (2020). More broadly, variable selection has been deployed as a tool for interpretable machine learning, including for highly complex and nonlinear models (e.g., Ribeiro et al., 2016; Afrabandpey et al., 2020).

Although often considered the “gold standard” of variable selection, subset selection remains underutilized due to several critical limitations. First, subset selection is inherently unstable: it is common to obtain entirely distinct subsets under perturbations or resampling of the data. This instability undermines the interpretability of a single “best” subset, and is a significant motivating factor for the proposed methods. Second, the solutions to (1) are unregularized. While it is advantageous to avoid *overshrinkage*, Hastie et al. (2020) showed that the lack of any regularization in (1) leads to deteriorating performance relative to penalized regression in low-signal settings. Third, inference about β requires careful adjustment for selection bias, which limits the available options for uncertainty quantification. Finally, solving (1) is computationally demanding even for moderate p, which has spawned many algorithmic advancements spanning multiple decades (e.g., Furnival and Wilson, 2000; Gatu and Kontoghiorghes, 2006; Bertsimas et al., 2016). For these reasons, penalized regression techniques that replace the $\ell_{0}$-penalty with convex or nonconvex yet computationally feasible alternatives (Fan and Lv, 2010) are often preferred.

From a Bayesian perspective, (1) is usually translated into a predictive model via a Gaussian (log-) likelihood and a sparsity (log-) prior. Indeed, substantial research efforts have been devoted to both sparsity (e.g., Ishwaran and Rao, 2005) and shrinkage (e.g., Polson and Scott, 2010) priors for β. Yet the prior alone cannot *select* subsets: the prior is the component of the data-generating process that incorporates prior beliefs, information, or regularization, while selection is ultimately a decision problem (Lindley, 1968). For instance, Barbieri and Berger (2004) and Liang et al. (2013) applied sparsity priors to obtain posterior inclusion probabilities, which were then used for *marginal* selection and screening, respectively. Jin and Goh (2020) selected subsets using marginal likelihoods, but required conjugate priors for Gaussian linear models. Hahn and Carvalho (2015) more fully embraced the decision analysis approach for variable selection, and augmented a squared error loss with an $\ell_{1}$-penalty for linear variable selection. Alternative loss functions have been proposed for seemingly unrelated regressions (Puelz et al., 2017), graphical models (Bashir et al., 2019), nonlinear regressions (Woody et al., 2020), functional regression (Kowal and Bourgeois, 2020), time-varying parameter models (Huber et al., 2020), and a variety of Kullback-Leibler approximations to the likelihood (Goutis and Robert, 1998; Nott and Leng, 2010; Tran et al., 2012; Piironen et al., 2020).

Despite the appropriate deployment of decision analysis for selection, each of these Bayesian methods relies on $\ell_{1}$-penalization or forward search. As such, they are restricted to limited search paths that cannot fully solve the (exhaustive) subset selection problem. More critically, these Bayesian approaches—as well as most classical ones—are unified in their emphasis on selecting a *single* “best” subset. However, in practice it is common for many subsets (or models) to achieve near-optimal predictive performance, known as the *Rashomon effect* (Breiman, 2001). This effect is particularly pronounced for correlated covariates, weak signals, or small sample sizes. Under these conditions, it is empirically and theoretically possible for the “true” covariates to be predictively inferior to a proper subset (Wu et al., 2007). As a result, the “best” subset is not only less valuable but also less interpretable. Reporting a single subset—or a small number of subsets along a highly restricted search path—obscures the likely presence of many distinct yet equally-predictive subsets of variables.

We advance an agenda that instead curates and summarizes a *family* of optimal or near-optimal subsets. This broader analysis alleviates the instability issues of a single “best” subset and provides a more complete predictive picture. The proposed approach operates within a decision analysis framework and is compatible with *any* Bayesian model ℳ for prediction or classification. Naturally, ℳ should represent the modeler’s beliefs about the data-generating process and describe the salient features in the data. Several key developments are required:

1. We derive *optimal* (in a decision analysis sense) linear coefficients for any subset of variables.
  Crucially, these coefficients inherit regularization *and* uncertainty quantification via ℳ, but avoid the overshrinkage induced by $\ell_{1}$-penalization. As such, these point estimators resolve multiple limitations of classical subset selection and ($\ell_{1}$-penalized) Bayesian decision-analytic variable selection, and further are adapted to both regression and classification problems. Next,
2. We design a modified branch-and-bound algorithm for *efficient exploration* over the space of candidate subsets.
  The search process is a vital component of subset selection, and our modular framework is broadly compatible with other state-of-the-art search algorithms (e.g., Bertsimas et al., 2016). Until now, these algorithms have not been fully deployed or adapted for Bayesian subset selection. Additionally,
3. We leverage the predictive distribution under ℳ to collect the *acceptable family* of near-optimal subsets.
  A core feature of the acceptable family is that it is defined using out-of-sample metrics and predictive uncertainty quantification, yet is computed using *in-sample posterior functionals* from a single model fit of ℳ. Hence, we maintain computational scalability and coherent uncertainty quantification that avoids data reuse. Lastly,
4. We *summarize* the acceptable family with key member subsets, such as the “best” (in terms of cross-validation error) predictor and the smallest acceptable subset, along with new (co-) variable importance metrics that measure the frequency with which variables (co-) appear in all, some, or no acceptable subsets.
  Unlike variable importances based on effect size, this inclusion-based metric effectively measures how many “predictively plausible” explanations (i.e., near-optimal subsets) contain each (pair of) covariate(s) as a member. Notably, each of these developments is presented for both prediction and classification.

The importance of curating and exploring a collection of subsets has been acknowledged previously. Existing approaches are predominantly frequentist, including fence methods (Jiang et al., 2008), Rashomon sets (Semenova and Rudin, 2019), bootstrapped confidence sets (Lei, 2019), and subsampling-based forward selection (Kissel and Mentch, 2021). Although the acceptable family has appeared previously for Bayesian decision analysis (Kowal, 2021; Kowal et al., 2021), it was applied only along the $\ell_{1}$-path which does not enumerate a sufficiently rich collection of competitive subsets. Further, previous applications of the acceptable family did not address points 1., 2., and 4. above.

The paper is outlined as follows. Section 2 contains the Bayesian subset search procedures, the construction of acceptable families, the (co-) variable importance metrics, and the predictive uncertainty quantification. Section 3 details the simulation study. The methodology is applied to a large education dataset in Section 4. We conclude in Section 5. The Appendix provides additional algorithmic details, simulation studies, and results from the application. An R package is available online at https://github.com/drkowal/ BayesSubsets. Although the education dataset (Children’s Environmental Health Initiative, 2020) cannot be released due to privacy protections, access to the dataset can occur through establishing affiliation with the Children’s Environmental Health Initiative (contact cehi@nd.edu).

## 2. Methods

### 2.1. Predictive decision analysis

Decision analysis establishes the framework for extracting actions, estimators, and predictions from a Bayesian model (e.g., Bernardo and Smith, 2009). These tools translate probabilistic models into practical decision-making and can be deployed to summarize or interpret complex models. However, additional methodology is needed to convert subset selection into a decision problem, and further to evaluate and collect many near-optimal subsets.

Let ℳ denote any Bayesian model with a proper posterior distribution $p_{ℳ}\left(\theta |y\right)$ and posterior predictive distribution

$$p_{ℳ}\left(\overset{˜}{y}|y\right)≔\int p_{ℳ}\left(\overset{˜}{y}|\theta ,y\right)p_{ℳ}\left(\theta |y\right)d\theta ,$$

where θ denotes the parameters of the model ℳ and y is the observed data. Informally, $p_{ℳ}\left(\overset{˜}{y}|y\right)$ defines the distribution of future or unobserved data $\overset{˜}{y}$ conditional on the observed data y and according to the model ℳ. Decision analysis evaluates each *action δ* based on a loss function $ℒ\left(\overset{˜}{y},\delta \right)$ that enumerates the cost of each action when $\overset{˜}{y}$ is realized. Examples include point prediction (e.g., squared error loss) or classification (e.g., cross-entropy loss), interval estimation (e.g., minimum length subject to 1−α coverage), and selection among a set of hypotheses (e.g., 0–1 loss). Since $\overset{˜}{y}$ is unknown yet modeled probabilistically under ℳ, an optimal action minimizes the posterior predictive expected loss

(2) $$\hat{\delta }≔\text{arg }\underset{\delta }{\text{min}}\;E_{\overset{˜}{y}|y}ℒ\left(\overset{˜}{y},\delta \right)$$

with the expectation taken under the Bayesian model ℳ. The operation in (2) averages the predictive loss over the possible realizations of $\overset{˜}{y}$ according to the posterior probability under ℳ and then minimizes the resulting quantity over the action space.

Yet without careful specification of the loss function ℒ, (2) does not provide a clear pathway for subset selection. To see this, let $\overset{˜}{y}\left(x\right)$ denote the predictive variable at covariate x. For point prediction of $\overset{˜}{y}\left(x\right)$ under squared error loss, the optimal action is

$$\overset{ˆ}{\delta }\left(x\right)≔\text{arg }\underset{\delta }{\text{min }}E_{\overset{˜}{y}|y}∥\overset{˜}{y}\left(x\right)-\delta \left(x\right){∥}_{2}^{2}\;=E_{\overset{˜}{y}|y}\left\{\tilde{y}\left(x\right)\right\},$$

i.e., the posterior predictive expectation at x. Similarly, for classification of $\overset{˜}{y}\left(x\right)\in \left\{0,1\right\}$ under cross-entropy loss (see (10)), the optimal action is the posterior predictive probability $\overset{ˆ}{\delta }\left(x\right)=p_{ℳ}\left\{\overset{˜}{y}\left(x\right)=1|y\right\}$. For a generic model ℳ, there is not necessarily a closed form for $\overset{ˆ}{\delta }\left(x\right)$ : these actions are computed separately for each x with no clear mechanism for inducing sparsity or specifying distinct subsets. Hence, additional techniques are needed to supply actions that are not only optimal but also selective and interpretable.

Note that we use observation-driven rather than parameter-driven loss functions. Unlike the parameters θ, which are unobservable and model-specific, the predictive variables $\overset{˜}{y}$ are observable and directly comparable across distinct Bayesian models. A decision analysis based on $\overset{˜}{y}$ operates on the same scale and in the same units as the data that have been—and will be—observed, which improves interpretability. Perhaps most important, an observation-driven decision analysis enables empirical evaluation of the selected actions.

### 2.2. Subset search for linear prediction

The subset search procedure is built within the decision framework of (2). For any Bayesian model ℳ, we consider *linear* actions δ(x)=x′δ with $\delta \in {\mathbb{R}}^{p}$, which offer both interpretability and the capacity for selection. Let $\delta_{𝒮}$ denote the linear action with zero coefficients for all j∉𝒮, where 𝒮⊆{1,…,p} is a subset of active variables. For prediction, we assemble the aggregate and weighted squared error loss

(3) $$ℒ\left({\left\{{\tilde{y}}_{i}\right\}}_{i=1}^{\tilde{n}},\delta_{𝒮}\right)=\overset{\tilde{n}}{\underset{i=1}{\sum }}\omega \left({\overset{˜}{x}}_{i}\right){\left\|{\tilde{y}}_{i}-{\overset{˜}{x}}_{i}^{\prime }\delta_{𝒮}\right\|}_{2}^{2}$$

where ${\tilde{y}}_{i}≔\overset{˜}{y}\left({\overset{˜}{x}}_{i}\right)$ is the predictive variable at ${\overset{˜}{x}}_{i}$ for each $i=1,\ldots ,\tilde{n}$ and $\omega \left({\overset{˜}{x}}_{i}\right)>0$ is a weighting function. The covariate values ${\left\{{\overset{˜}{x}}_{i}\right\}}_{i=1}^{\tilde{n}}$ can be distinct from the observed covariate values ${\left\{x_{i}\right\}}_{i=1}^{n}$, for example to evaluate the action for a different population.

The loss in (3) evaluates linear coefficients $\delta_{𝒮}$ for any given subset 𝒮 by accumulating the squared error loss over the covariate values ${\left\{{\overset{˜}{x}}_{i}\right\}}_{i=1}^{\tilde{n}}$. Since (3) depends on ${\left\{{\tilde{y}}_{i}\right\}}_{i=1}^{\tilde{n}}$, the loss inherits a joint predictive distribution $p_{ℳ}\left({\tilde{y}}_{1},\ldots ,{\tilde{y}}_{\tilde{n}}|y\right)$ from ℳ. The loss is decoupled from the Bayesian model ℳ : the linear action does not require a linearity assumption for ℳ. The weights $\omega \left({\overset{˜}{x}}_{i}\right)$ can be used to target actions locally, which provides a sparse and local linear approximation to ℳ. For example, we might parametrize the weighting function as $\omega \left({\overset{˜}{x}}_{i}\right)\propto \text{exp}\left(-{\left\|{\overset{˜}{x}}_{i}-x^{\text{*}}\right\|}_{2}^{2}/\ell \right)$ with range parameter ℓ in order to weight based on proximity to some particular $x^{\text{*}}$ of interest (Ribeiro et al., 2016). Alternatively, ω can be specified via a probability model for the likelihood of observing each covariate value, including $\omega \left({\overset{˜}{x}}_{i}\right)={\tilde{n}}^{-1}$ as a simple yet useful example, especially when using the observed covariate values ${\left\{{\overset{˜}{x}}_{i}\right\}}_{i=1}^{\tilde{n}}={\left\{x_{i}\right\}}_{i=1}^{n}$; this is our default choice.

Crucially, the optimal action can be solved directly for any subset 𝒮:

**Lemma 1** *Suppose* $E_{\overset{˜}{y}|y}\overset{˜}{y}{\left({\overset{˜}{x}}_{i}\right)}_{2}^{2}<\infty$ for $i=1,\ldots ,\tilde{n}$. *The optimal action* (2) *for the loss* (3) *is given by the nonzero entries*

(4) $${\overset{ˆ}{\delta }}_{𝒮}=\text{arg }\underset{\delta_{𝒮}}{\text{min}}\overset{\tilde{n}}{\underset{i=1}{\sum }}\omega \left({\overset{˜}{x}}_{i}\right){\left\|{\hat{y}}_{i}-{\overset{˜}{x}}_{i}^{\prime }\delta_{𝒮}\right\|}_{2}^{2}$$

(5) $$={\left({\overset{˜}{X}}_{𝒮}^{\prime }\Omega {\overset{˜}{X}}_{𝒮}\right)}^{-1}{\overset{˜}{X}}_{𝒮}^{\prime }\Omega \overset{ˆ}{y},$$

*with zeros for indices* j∉𝒮, *where* ${\hat{y}}_{i}≔E_{\overset{˜}{y}|y}\left\{\overset{˜}{y}\left({\overset{˜}{x}}_{i}\right)\right\},\overset{ˆ}{y}≔{\left({\hat{y}}_{1},\ldots ,{\hat{y}}_{\tilde{n}}\right)}^{\prime },\Omega ≔\text{diag}{\left\{\omega \left({\overset{˜}{x}}_{i}\right)\right\}}_{i=1}^{\tilde{n}}$, *and ${\overset{˜}{X}}_{𝒮}$ is the* $\tilde{n}\times \left|𝒮\right|$ *matrix of the active covariates in* ${\left\{{\overset{˜}{x}}_{i}\right\}}_{i=1}^{\tilde{n}}$ *for subset* 𝒮.

**Proof** It is sufficient to observe that $E_{\overset{˜}{y}|y}{\left\|\overset{˜}{y}\left({\overset{˜}{x}}_{i}\right)-{\overset{˜}{x}}_{i}^{\text{'}}\delta_{𝒮}\right\|}_{2}^{2}=E_{\overset{˜}{y}|y}{\left\|\left\{\overset{˜}{y}\left({\overset{˜}{x}}_{i}\right)-{\hat{y}}_{i}\right\}+\left({\hat{y}}_{i}-{\overset{˜}{x}}_{i}^{\text{'}}\delta_{𝒮}\right)\right\|}_{2}^{2}=E_{\overset{˜}{y}|y}{\left\|\overset{˜}{y}\left({\overset{˜}{x}}_{i}\right)-{\hat{y}}_{i}\right\|}_{2}^{2}+{\left\|{\hat{y}}_{i}-{\overset{˜}{x}}_{i}^{\text{'}}\delta_{𝒮}\right\|}_{2}^{2}$, where the first term is a (finite) constant that does not depend on $\delta_{𝒮}$. The remaining steps constitute a weighted least squares solution. ■

The consequence of Lemma 1 is that the optimal action for each subset 𝒮 is simply the (weighted) least squares solution based on pseudo-data $\left({\overset{˜}{X}}_{𝒮},\overset{ˆ}{y}\right)$—i.e., a “fit to the fit” from ℳ. The advantages of ℳ can be substantial: the Bayesian model propagates regularization (e.g., shrinkage, sparsity, or smoothness) to the point predictions $\overset{ˆ}{y}$, which typically offers sizable improvements in estimation and prediction relative ordinary (weighted) least squares. This effect is especially pronounced in the presence of high-dimensional (large p) or correlated covariates. The optimal action may be non-unique if ${\overset{˜}{X}}_{𝒮}^{\prime }\Omega {\overset{˜}{X}}_{𝒮}$ is noninvertible, in which case the inverse in (5) can be replaced by a generalized inverse.

At this stage, the Bayesian model ℳ is only needed to supply the pseudo-response variable ${\hat{y}}_{i}$; different choices of ℳ will result in distinct values of ${\hat{y}}_{i}$ and therefore distinct actions ${\overset{ˆ}{\delta }}_{𝒮}$. An illuminating special case occurs for linear regression:

**Corollary 2** *For the linear regression model* ℳ *with* $E_{\overset{˜}{y}|\theta }\left\{\overset{˜}{y}\left(x\right)\right\}=x\prime \beta$ *and any set of covariate values* ${\left\{{\overset{˜}{x}}_{i}\right\}}_{i=1}^{\tilde{n}}$ *and weights* $\omega \left({\overset{˜}{x}}_{i}\right)>0$, *the optimal action* (2) *under* (3) *for the full set of covariates is* ${\overset{ˆ}{\delta }}_{\left\{1,\ldots ,p\right\}}=\overset{ˆ}{\beta }$, *where* $\overset{ˆ}{\beta }≔E_{\theta |y}\beta$.

Depending on the choice of ${\left\{{\overset{˜}{x}}_{i}\right\}}_{i=1}^{\tilde{n}}$ and $\omega ,{\overset{ˆ}{\delta }}_{\left\{1,\ldots ,p\right\}}$ may be non-unique. Corollary 2 links the optimal action to the model parameters: the posterior expectation $\overset{ˆ}{\beta }$ is also the optimal action under the parameter-driven squared error loss $ℒ\left(\beta ,\delta \right)=∥\beta -\delta {∥}_{2}^{2}$. Similarly, a linear model for ℳ implies that ${\hat{y}}_{i}={\overset{˜}{x}}_{i}^{\prime }\overset{ˆ}{\beta }$, so the optimal action (5) for any subset 𝒮 is intrinsically connected to the (regression) model parameters. This persists for other regression models as well. By contrast, these restrictions also illustrate the generality of (3)-(5): the optimal linear actions are derived explicitly under any model ℳ (with $E_{\overset{˜}{y}|y}{\left\|\overset{˜}{y}\left({\overset{˜}{x}}_{i}\right)\right\|}_{2}^{2}<\infty$) and using any set of covariate values ${\left\{{\overset{˜}{x}}_{i}\right\}}_{i=1}^{\tilde{n}}$, active covariates 𝒮⊆{1,…,p}, and weighting functions $\omega \left(\overset{˜}{x}\right)>0$.

The critical remaining challenge is optimization—or at least evaluation and comparison—among the possible subsets 𝒮. Our strategy emerges from the observation that there may be many subsets that achieve near-optimal predictive performance, often referred to as the *Rashomon effect* (Breiman, 2001). The goal is to collect, characterize, and compare these near-optimal subsets of linear predictors. Hence, there are two core tasks: (i) identify candidate subsets and (ii) filter to include only those subsets that achieve near-optimal predictive performance. These tasks must overcome both computational and methodological challenges—similar to classical (non-Bayesian) subset selection—which we resolve in the subsequent sections.

An exhaustive enumeration of all possible subsets presents an enormous computational burden, even for moderate p. Although tempting, it is misguided to consider direct optimization over all possible subsets of {1,…,p},

(6) $${\overset{ˆ}{\delta }}_{\hat{𝒮}}≔\text{arg }\underset{𝒮,\delta_{𝒮}}{\text{min }}E_{\overset{˜}{y}|y}ℒ\left({\left\{{\tilde{y}}_{i}\right\}}_{i=1}^{\tilde{n}},\delta_{𝒮}\right),$$

for the aggregate squared error loss (3). To see this—and find suitable alternatives—consider the following result:

**Lemma 3** *Let* $RSS\left(y,\mu \right)≔∥y-\mu {∥}_{2}^{2}$ *and* $\overset{ˆ}{y}≔E_{\overset{˜}{y}|y}\overset{˜}{y}$, *and suppose* $E_{\overset{˜}{y}|y}∥\overset{˜}{y}{∥}_{2}^{2}<\infty$. *For any point predictors* $\mu_{1}$ *and* $\mu_{2}$, *we have*

(7) $$E_{\overset{˜}{y}|y}\left\{RSS\left(\overset{˜}{y},\mu_{1}\right)\right\}\leq E_{\overset{˜}{y}|y}\left\{RSS\left(\overset{˜}{y},\mu_{2}\right)\right\}\Leftrightarrow RSS\left(\overset{ˆ}{y},\mu_{1}\right)\leq RSS\left(\overset{ˆ}{y},\mu_{2}\right).$$

**Proof** Since $E_{\overset{˜}{y}|y}\left\{\text{RSS}\left(\overset{˜}{y},\mu \right)\right\}-\text{RSS}\left(\overset{ˆ}{y},\mu \right)=E_{\overset{˜}{y}|y}∥\overset{˜}{y}{∥}_{2}^{2}-∥\overset{ˆ}{y}{∥}_{2}^{2}$ is finite and does not depend on μ, the ordering of $\mu_{1}$ and $\mu_{2}$ will be identical whether using $E_{\overset{˜}{y}|y}\left\{\text{RSS}\left(\overset{˜}{y},\mu \right)\right\}$ or $\text{RSS}\left(\overset{ˆ}{y},\mu \right)$. ■

Notably, $E_{\overset{˜}{y}|y}\left\{\text{RSS}\left(\overset{˜}{y},\mu \right)\right\}$ is the key constituent in optimizing the predictive squared error loss (3), while $\text{RSS}\left(\overset{ˆ}{y},\mu \right)$ is simply the usual residual sum-of-squares (RSS) with $\overset{ˆ}{y}$ replacing y.

Now recall the optimization of (6). For any subset 𝒮, the optimal action is the least squares solution (5) with pseudo-data $\overset{ˆ}{y}$. However, RSS in linear regression is ordered by nested subsets: $\text{RSS}\left(\overset{ˆ}{y},\overset{˜}{X}{\overset{ˆ}{\delta }}_{{𝒮}_{1}}\right)\leq \text{RSS}\left(\overset{ˆ}{y},\overset{˜}{X}{\overset{ˆ}{\delta }}_{{𝒮}_{2}}\right)$ whenever ${𝒮}_{2}\subseteq {𝒮}_{1}$. By Lemma 3, it follows that the solution of (6) is simply

$${\overset{ˆ}{\delta }}_{\hat{𝒮}}={\left({\overset{˜}{X}}^{\prime }\Omega \overset{˜}{X}\right)}^{-1}{\overset{˜}{X}}^{\prime }\Omega \overset{ˆ}{y},\;\hat{𝒮}=\left\{1,\ldots ,p\right\}$$

for $\overset{˜}{X}={\left({\overset{˜}{x}}_{1},\ldots ,{\overset{˜}{x}}_{\tilde{n}}\right)}^{\prime }$. As with (5), a generalized inverse can be substituted if necessary. The main consequence is that the optimal actions in (4) and (6) alone cannot select variables or subsets: (4) provides the optimal action for a given subset 𝒮, while (6) trivially returns the full set of covariates. Despite the posterior predictive expectation in (6), this optimality is only valid in-sample and is unlikely to persist for out-of-sample prediction. Hence, this optimality is unsatisfying.

Yet Lemma 3 provides a path forward. Rather than fixing a subset 𝒮 in (4) or optimizing over all subsets in (6), suppose we compare among all subsets up to size k≤p. Equivalently, this constraint can be representation as an $\ell_{0}$-penalty augmentation to the loss function (3), i.e.,

(8) $${\overset{ˆ}{\delta }}_{k}≔\text{arg }\underset{𝒮,\delta_{𝒮}}{\text{min }}E_{\overset{˜}{y}|y}ℒ\left({\left\{{\tilde{y}}_{i}\right\}}_{i=1}^{\tilde{n}},\delta_{𝒮}\right)\text{ subject to }{\left\|\delta_{𝒮}\right\|}_{0}\leq k.$$

In direct contrast with previous approaches for Bayesian variable selection via decision analysis (e.g., Hahn and Carvalho, 2015; Woody et al., 2020; Kowal et al., 2021) we do *not* use convex relaxations to $\ell_{1}$-penalties, which create unnecessarily restrictive search paths and introduce additional bias in the coefficient estimates.

Under the loss (3), the solution to (8) reduces to

(9) $${\overset{ˆ}{\delta }}_{k}=\text{arg }\underset{𝒮,\delta_{𝒮}}{\text{min }}\overset{\tilde{n}}{\underset{i=1}{\sum }}\omega \left({\overset{˜}{x}}_{i}\right){\left\|{\hat{y}}_{i}-{\overset{˜}{x}}_{i}^{\prime }\delta_{𝒮}\right\|}_{2}^{2}\text{ subject to }{\left\|\delta_{𝒮}\right\|}_{0}=k$$

using the “fit-to-the-fit” arguments from Lemma 1 and the RSS ordering results from Lemma 3. In particular, the solution ${\overset{ˆ}{\delta }}_{k}$ resembles classical subset selection (1), but uses the fitted values ${\hat{y}}_{i}$ from ℳ instead of the data y and further generalizes to include possibly distinct covariates $\left\{{\overset{˜}{x}}_{i}\right\}$ and weights $\left\{\omega \left({\overset{˜}{x}}_{i}\right)\right\}$.

Because of the representation in (9), we can effectively solve (8) by leveraging existing algorithms for subset selection. However, our broader interest in the collection of near-optimal subsets places greater emphasis on the search and filtering process. For any two subsets ${𝒮}_{1}$ and ${𝒮}_{2}$ of equal size $k=\left|{𝒮}_{1}\right|=\left|{𝒮}_{2}\right|$, Lemma 3 implies that $E_{\overset{˜}{y}|y}\left\{\text{RSS}\left(\overset{˜}{y},\overset{˜}{X}\delta_{{𝒮}_{1}}\right)\right\}\leq E_{\overset{˜}{y}|y}\left\{\text{RSS}\left(\overset{˜}{y},\overset{˜}{X}\delta_{{𝒮}_{2}}\right)\right\}$ if and only if $\text{RSS}\left(\overset{ˆ}{y},\overset{˜}{X}\delta_{{𝒮}_{1}}\right)\leq \text{RSS}\left(\overset{ˆ}{y},\overset{˜}{X}\delta_{{𝒮}_{2}}\right)$. Therefore, we can order the linear actions from (4) among all equally-sized subsets simply by ordering the values of $\text{RSS}\left(\overset{ˆ}{y},\overset{˜}{X}\delta_{𝒮}\right)$. This result resembles the analogous scenario in classical linear regression on ${\left\{\left(x_{i},y_{i}\right)\right\}}_{i=1}^{n}$ : subsets of fixed size maintain the same ordering whether using RSS or information criteria such as AIC, BIC, or Mallow’s $C_{p}$. Here, the criterion of interest is the posterior predictive expected RSS, $E_{\overset{˜}{y}|y}\left\{\text{RSS}\left(\overset{˜}{y},\overset{˜}{X}\delta_{𝒮}\right)\right\}$, and the RSS reduction occurs with the model ℳ fitted values ${\hat{y}}_{i}=E_{\overset{˜}{y}|y}\overset{˜}{y}\left({\overset{˜}{x}}_{i}\right)$ serving as pseudo-data.

Because of this RSS-based ordering among equally-sized linear actions, we can leverage the computational advantages of the *branch-and-bound algorithm* (BBA) for efficient subset exploration (Furnival and Wilson, 2000). Using a tree-based enumeration of all possible subsets, BBA avoids an exhaustive subset search by carefully eliminating non-competitive subsets (or branches) according to RSS. BBA is particularly advantageous for (i) selecting $s_{max}\leq p$ covariates, (ii) filtering to $m_{k}\leq \left(\begin{array}{c} p\\ k \end{array}\right)$ subsets for each size k, and (iii) exploiting the presence of covariates that are almost always present for low-RSS models (Miller, 1984). In the present setting, the key inputs to the algorithm are the covariates $\left\{{\overset{˜}{x}}_{i}\right\}$, the pseudo-data $\left\{{\hat{y}}_{i}\right\}$, and the weights $\left\{\omega \left({\overset{˜}{x}}_{i}\right)\right\}$. In addition, we specify the maximum number of subsets $m_{k}\leq \left(\begin{array}{l} p\\ k \end{array}\right)$ to return for each size k. As $m_{k}$ increases, BBA returns more subsets—with higher RSS—yet computational efficiency deteriorates. We consider default values of $m_{k}=100$ and $m_{k}=15$ and compare the results in Section 4. An efficient implementation of BBA is available in the leaps package in R. Note that our framework is also compatible with many other subset search algorithms (e.g., Bertsimas et al., 2016).

In the case of moderate to large p, we screen to $s_{max}\leq p$ covariates using the original model ℳ. Specifically, we select the $s_{max}$ covariates with the largest absolute (standardized) linear regression coefficients. When ℳ is a nonlinear model, we use the optimal linear coefficients ${\overset{ˆ}{\delta }}_{𝒮}$ on the full subset 𝒮={1,…,p} of (standardized) covariates. The use of marginal screening is common in both frequentist (Fan and Lv, 2008) and Bayesian (Bondell and Reich, 2012) high-dimensional linear regression models, with accompanying consistency results in each case. Here, sceening is motivated by computational scalability and interpretability: BBA is quite efficient for p≤35 and $m_{k}\leq 100$, while the interpretation of a subset of covariates—acting jointly to predict accurately—is muddled as the subset size increases. By default, we fix $s_{max}=35$. We emphasize that although this screening procedure relies on marginal criteria, it is based on a joint model ℳ that incorporates all p covariates. In that sense, our *screening* procedure resembles the most popular Bayesian variable *selection* strategies based on posterior inclusion probabilities (Barbieri and Berger, 2004) or hard-thresholding (Datta and Ghosh, 2013).

### 2.3. Subset search for logistic classifiers

Classification and binary regression operate on {0, 1}, rendering the squared error loss (3) unsuitable. Consider a binary predictive functional $h\left(\tilde{y}\right)\in \left\{0,1\right\}$ where $\tilde{y}\sim p_{ℳ}\left(\tilde{y}|y\right)$. In this framework, binarization can come from one of two sources. Most common, the data are binary $y_{i}\in \left\{0,1\right\}$, paired with the identity functional $h\left(\tilde{y}\right)=\tilde{y}$, and ℳ is a Bayesian classification (e.g., probit or logistic regression) model. Less common, non-binary data can be modeled via ℳ and paired with a functional h that maps to {0, 1}.For example, we may be interested in selecting variables to predict exceedance of a threshold, $h\left(\tilde{y}\right)=I\left\{\tilde{y}\geq \tau \right\}$, for some τ based on real-valued data y. The latter case is an example of *targeted prediction* (Kowal, 2021), which customizes posterior summaries or decisions for any functional h. This approach is distinct from fitting separate models to each empirical functional ${\left\{h\left(y_{i}\right)\right\}}_{i=1}^{n}$—which is still compatible with the first setting above—and instead requires only a single Bayesian reference model ℳ for all target functionals h.

For classification or binary prediction of $h\left(\tilde{y}\right)\in \left\{0,1\right\}$, we replace the squared error loss (3) with the aggregate and weighted cross-entropy loss,

(10) $$ℒ\left[{\left\{h\left({\tilde{y}}_{i}\right)\right\}}_{i=1}^{\tilde{n}},\delta_{𝒮}\right]=\overset{\tilde{n}}{\underset{i=1}{\sum }}\omega \left({\overset{˜}{x}}_{i}\right)\left[h\left({\tilde{y}}_{i}\right)\text{log}\left\{\pi_{𝒮}\left({\overset{˜}{x}}_{i}\right)\right\}+\left\{1-h\left({\tilde{y}}_{i}\right)\right\}\text{log}\left\{1-\pi_{𝒮}\left({\overset{˜}{x}}_{i}\right)\right\}\right],$$

where $h\left({\tilde{y}}_{i}\right)\in \left\{0,1\right\}$ is the predictive variable at ${\overset{˜}{x}}_{i}$ under ℳ and $\pi_{𝒮}\left({\overset{˜}{x}}_{i}\right)≔{\left\{1+\text{exp}\left(-{\overset{˜}{x}}_{i}^{\prime }\delta_{𝒮}\right)\right\}}^{-1}$. The cross-entropy is also the *deviance* or negative log-likelihood of a series of independent Bernoulli random variables $h\left({\tilde{y}}_{i}\right)$ each with success probability $\pi_{𝒮}\left({\overset{˜}{x}}_{i}\right)$ for $i=1,\ldots ,\tilde{n}$. However, (10) does not imply a distributional assumption for the decision analysis; all distributional assumptions are encapsulated within ℳ, including the posterior predictive distribution of $h\left({\tilde{y}}_{i}\right)$. As before, $\delta_{𝒮}$ is the linear action with zero coefficients for all j∉𝒮, where 𝒮⊆{1,…,p} is a subset of active variables.

The optimal action (2) is obtained for each subset 𝒮 by computing expectations with respect to the posterior predictive distribution under ℳ and minimizing the ensuing quantity. As in the case of squared error loss, key simplifications are available:

(11) $${\overset{ˆ}{\delta }}_{𝒮}=\text{arg }\underset{\delta_{𝒮}}{\text{min }}\overset{\tilde{n}}{\underset{i=1}{\sum }}\omega \left({\overset{˜}{x}}_{i}\right)\left[{\hat{h}}_{i}\text{log}\left\{\pi_{𝒮}\left({\overset{˜}{x}}_{i}\right)\right\}+\left(1-{\hat{h}}_{i}\right)\text{log}\left\{1-\pi_{𝒮}\left({\overset{˜}{x}}_{i}\right)\right\}\right]$$

where ${\hat{h}}_{i}≔E_{\overset{˜}{y}|y}h\left({\tilde{y}}_{i}\right)$ is the posterior predictive expectation of $h\left({\tilde{y}}_{i}\right)$ under ℳ. The representation in (11) is quite useful: it is the negative log-likelihood for a logistic regression model with pseudo-data ${\left\{\left({\overset{˜}{x}}_{i},{\hat{h}}_{i}\right)\right\}}_{i=1}^{\tilde{n}}$. Standard algorithms and software, such as iteratively reweighted least squares (IRLS) in the glm package in R, can be applied to solve (11) for any subset 𝒮.

Instead of fitting a logistic regression to the observed binary variables $h\left(y_{i}\right)\in \left\{0,1\right\}$, the optimal action under cross-entropy (10) fits to the posterior predictive probabilities ${\hat{h}}_{i}=p_{ℳ}\left\{h\left({\tilde{y}}_{i}\right)=1|y\right\}\in \left[0,1\right]$ under ℳ. For a well-specified model ℳ, these posterior probabilities ${\hat{h}}_{i}$ can be more informative than the binary empirical functionals $h\left(y_{i}\right)$ : the former lie on a continuum between the endpoints zero and one. Furthermore, for non-degenerate models ℳ with ${\hat{h}}_{i}\in \left(0,1\right)$, the optimal action (11) resolves the issue of separability, which is a persistent challenge in classical logistic regression.

Despite the efficiency of IRLS for a fixed subset 𝒮, the computational savings of BBA for subset search rely on the RSS from a linear model. As such, solving (11) for all or many subsets incurs a much greater computational cost. Yet IRLS is intrinsically linked with RSS. At convergence, IRLS obtains a weighted least squares solution

(12) $${\overset{ˆ}{\delta }}_{𝒮}={\left({\overset{˜}{X}}_{𝒮}^{\prime }W{\overset{˜}{X}}_{𝒮}\right)}^{-1}{\overset{˜}{X}}_{𝒮}^{\prime }Wz$$

where $W≔\text{diag}{\left\{w_{i}\right\}}_{i=1}^{\tilde{n}}$ for weights $w_{i}≔\omega \left({\overset{˜}{x}}_{i}\right){\hat{\pi }}_{𝒮}\left({\overset{˜}{x}}_{i}\right)\left\{1-{\hat{\pi }}_{𝒮}\left({\overset{˜}{x}}_{i}\right)\right\}$, fitted probabilities ${\hat{\pi }}_{𝒮}\left({\overset{˜}{x}}_{i}\right)≔{\left\{1+\text{exp}\left(-{\overset{˜}{x}}_{i}^{\prime }{\overset{ˆ}{\delta }}_{𝒮}\right)\right\}}^{-1}$, and pseudo-data

(13) $$z_{i}≔\text{log}\frac{{\hat{\pi }}_{𝒮}\left({\overset{˜}{x}}_{i}\right)}{1-{\hat{\pi }}_{𝒮}\left({\overset{˜}{x}}_{i}\right)}+\frac{{\hat{h}}_{i}-{\hat{\pi }}_{𝒮}\left({\overset{˜}{x}}_{i}\right)}{{\hat{\pi }}_{𝒮}\left({\overset{˜}{x}}_{i}\right)\left\{1-{\hat{\pi }}_{𝒮}\left({\overset{˜}{x}}_{i}\right)\right\}}$$

with $z≔{\left(z_{1},\ldots ,z_{\tilde{n}}\right)}^{\prime }$. By design, the weighted least squares objective associated with (12) is a second-order Taylor approximation to the predictive expected cross-entropy loss:

(14) $$E_{\overset{˜}{y}|y}ℒ\left[{\left\{h\left({\tilde{y}}_{i}\right)\right\}}_{i=1}^{\tilde{n}},{\overset{ˆ}{\delta }}_{𝒮}\right]\approx \overset{\tilde{n}}{\underset{i=1}{\sum }}\omega \left({\overset{˜}{x}}_{i}\right)\frac{{\left\{{\hat{h}}_{i}-{\hat{\pi }}_{𝒮}\left({\overset{˜}{x}}_{i}\right)\right\}}^{2}}{{\hat{\pi }}_{𝒮}\left({\overset{˜}{x}}_{i}\right)\left\{1-{\hat{\pi }}_{𝒮}\left({\overset{˜}{x}}_{i}\right)\right\}}=\overset{\tilde{n}}{\underset{i=1}{\sum }}w_{i}{\left(z_{i}-{\overset{˜}{x}}_{i}^{\prime }{\overset{ˆ}{\delta }}_{𝒮}\right)}^{2}.$$

The weighted least squares approximation in (14) summons BBA for subset search. Hosmer et al. (1989) adopted this strategy for subset selection in classical logistic regression on $y_{i}\in \left\{0,1\right\}$. Here, both the goals and the optimization criterion are distinct: we are interested in curating a collection of near-optimal subsets—rather than selecting a single “best” subset—and the weighted least squares objective (14) inherits the fitted probabilities ${\hat{h}}_{i}$ from the Bayesian model ℳ along with the weights $\omega \left({\overset{˜}{x}}_{i}\right)$.

Ideally, we might apply BBA directly based on the covariates $\left\{{\overset{˜}{x}}_{i}\right\}$, the pseudo-data $\left\{z_{i}\right\}$, and the weights $\left\{w_{i}\right\}$. However, both $z_{i}$ and $w_{i}$ depend on ${\hat{\pi }}_{𝒮}\left({\overset{˜}{x}}_{i}\right)$ and therefore are subset-specific. As a result, BBA cannot be applied without significant modifications. A suitable alternative is to construct subset-invariant psuedo-data and weights by replacing ${\hat{\pi }}_{𝒮}\left({\overset{˜}{x}}_{i}\right)$ with the corresponding estimate from the full model, ${\hat{h}}_{i}$. Specifically, let

(15) $${\hat{z}}_{i}≔\text{log}\left\{{\hat{h}}_{i}/\left(1-{\hat{h}}_{i}\right)\right\},\;{\hat{w}}_{i}≔\omega \left({\overset{˜}{x}}_{i}\right){\hat{h}}_{i}\left(1-{\hat{h}}_{i}\right),$$

both of which depend on ℳ rather than the individual subsets 𝒮. The pseudo-data ${\hat{z}}_{i}$ is defined similarly to $z_{i}$ in (13), where the second term now cancels. Finally, BBA subset search can be applied using the covariates $\left\{{\overset{˜}{x}}_{i}\right\}$, the pseudo-data $\left\{{\hat{z}}_{i}\right\}$, and the weights $\left\{{\hat{w}}_{i}\right\}$. As for squared error, we restrict each subset size to $m_{k}=100$ or $m_{k}=15$ for all subsets of size k=1,…,p. Despite the critical role of the weighted least squares approximation in (14) for subset search, all further evaluations and comparisons rely on the exact cross-entropy loss (10).

### 2.4. Predictive evaluations for identifying near-optimal subsets

The BBA subset search filters the $2^{p}$ possible subsets to the $m_{k}$ best subsets for each size $k=1,\ldots ,s_{\text{max}}$ according to posterior predictive expected loss using the weighted squared error loss for prediction (Section 2.2) or the cross-entropy loss for classification (Section 2.3). However, as noted below Lemma 3, further comparisons based on these expected losses are trivial: the full set 𝒮={1,…,p} always obtains the minimum, which precludes variable selection. Selection of a single “best” subset of each size k invites additional difficulties: if multiple subsets perform similarly—which is common for correlated covariates—then selecting $m_{k}=1$ subset will not be robust or stable against perturbations of the data. Equally important, restricting to $m_{k}=1$ subset is blind to competing subsets that offer similar predictive performance yet may differ substantially in the composition of covariates; see Sections 3–4. These challenges persist for both classical and Bayesian approaches.

We instead curate and explore a collection of near-optimal subsets. The notion of “near-optimal” derives from the *acceptable family* of Kowal (2021). Informally, the acceptable family uses out-of-sample predictive metrics to gather those predictors that match or nearly match the performance of the best out-of-sample predictor with nonnegligible posterior predictive probability under ℳ. The out-of-sample evaluation uses a modified K-fold cross-validation procedure. Let ${ℐ}_{k}\subset \left\{1,\ldots ,n\right\}$ denote the kth validation set, where each data point appears in (at least) one validation set, $\cup_{k=1}^{K}{ℐ}_{k}=\left\{1,\ldots ,n\right\}$. By default, we use K=10 validation sets that are equally-sized, mutually exclusive, and selected randomly from {1,…,n}. Define an evaluative loss function $L\left(y,{\overset{˜}{x}}^{\prime }{\overset{ˆ}{\delta }}_{𝒮}\right)$ for the optimal linear coefficients of subset 𝒮, and let S denote the collection of subsets obtained from the BBA filtering process. Typically, the evaluative loss L is identical to the loss ℒ used for optimization, but this restriction is not required. For each data split k and each subset 𝒮∈S, the out-of-sample *empirical* and *predictive* losses are

(16) $$L_{𝒮}^{out}\left(k\right)≔\frac{1}{\left|{ℐ}_{k}\right|}\underset{i\in {ℐ}_{k}}{\sum }L\left(y_{i},x_{i}^{\prime }{\overset{ˆ}{\delta }}_{𝒮}^{-{ℐ}_{k}}\right),\;{\tilde{L}}_{𝒮}^{out}\left(k\right)≔\frac{1}{\left|{ℐ}_{k}\right|}\underset{i\in {ℐ}_{k}}{\sum }L\left({\tilde{y}}_{i}^{-{ℐ}_{k}},x_{i}^{\prime }{\overset{ˆ}{\delta }}_{𝒮}^{-{ℐ}_{k}}\right)$$

respectively, where ${\overset{ˆ}{\delta }}_{𝒮}^{-{ℐ}_{k}}≔{\text{arg min}}_{\delta_{𝒮}}E_{\overset{˜}{y}|y^{-{ℐ}_{k}}}ℒ\left({\left\{{\tilde{y}}_{i}\right\}}_{i\notin {ℐ}_{k}},\delta_{𝒮}\right)$ is estimated using only the training data $y^{-{ℐ}_{k}}≔{\left\{y_{i}\right\}}_{i\notin {ℐ}_{k}}$ and ${\tilde{y}}_{i}^{-{ℐ}_{k}}\sim p_{ℳ}\left({\tilde{y}}_{i}|y^{-{ℐ}_{k}}\right)$ is the posterior predictive distribution at $x_{i}$ conditional only on the training data. Averaging across all data splits, we obtain $L_{𝒮}^{out}≔K^{-1}\sum_{k=1}^{K}L_{𝒮}^{out}\left(k\right)$ and ${\tilde{L}}_{𝒮}^{out}≔K^{-1}\sum_{k=1}^{K}{\tilde{L}}_{𝒮}^{out}\left(k\right)$.

The distinction between the empirical loss $L_{𝒮}^{out}$ and the predictive loss ${\tilde{L}}_{𝒮}^{out}$ is important. The empirical loss is a point estimate of the risk under predictions from $\delta_{𝒮}$ based on the data y. By comparison, the predictive loss provides a distribution of the out-of-sample loss based on the model ℳ. Both are valuable: $L_{𝒮}^{out}$ is entirely empirical and captures the classical notion of K-fold cross-validation, while ${\tilde{L}}_{𝒮}^{out}$ leverages the Bayesian model to propagate the uncertainty from the model-based data-generating process.

For any two subsets ${𝒮}_{1}$ and ${𝒮}_{2}$, consider the percent increase in out-of-sample predictive loss from ${\overset{ˆ}{\delta }}_{{𝒮}_{1}}$ to ${\overset{ˆ}{\delta }}_{{𝒮}_{2}}$:

(17) $${\tilde{D}}_{{𝒮}_{1},{𝒮}_{2}}^{out}≔100\times \left({\tilde{L}}_{{𝒮}_{2}}^{out}-{\tilde{L}}_{{𝒮}_{1}}^{out}\right)/{\tilde{L}}_{{𝒮}_{1}}^{out}.$$

Since ${\tilde{D}}_{{𝒮}_{1},{𝒮}_{2}}^{out}$ inherits a predictive distribution under ℳ, we can leverage the accompanying uncertainty quantification to determine whether the predictive performances of ${\overset{ˆ}{\delta }}_{{𝒮}_{1}}$ and ${\overset{ˆ}{\delta }}_{{𝒮}_{2}}$ are sufficiently distinguishable. In particular, we are interested in comparisons to the best subset for out-of-sample prediction,

(18) $${𝒮}_{min}≔\text{arg }\underset{𝒮\in S}{\text{min }}L_{𝒮}^{out},$$

so that ${\overset{ˆ}{\delta }}_{{𝒮}_{min}}$ is the optimal linear action associated with the subset ${𝒮}_{min}$ that minimizes the empirical K-fold cross-validated loss. Unlike the RSS-based in-sample evaluations from Section 2.2, the subset ${𝒮}_{min}$ can—and usually will—differ from the full set {1,…,p}, which enables variable selection driven by out-of-sample predictive performance.

Using ${𝒮}_{min}$ as an anchor, the acceptable family broadens to include near-optimal subsets:

(19) $$A_{\eta ,\varepsilon }≔\left\{𝒮\in S:{\mathbb{P}}_{ℳ}\left({\tilde{D}}_{{𝒮}_{min},𝒮}^{out}<\eta \right)\geq \varepsilon \right\},\;\eta \geq 0,\varepsilon \in \left[0,1\right].$$

With (19), we collect all subsets 𝒮 that perform within a margin η≥0% of the best subset, ${\tilde{D}}_{{𝒮}_{min},𝒮}^{out}<\eta$, with probability at least ε∈[0,1]. Equivalently, a subset 𝒮 is acceptable if and only if there exists a lower (1−ε) posterior prediction interval for ${\tilde{D}}_{{𝒮}_{min},𝒮}^{out}$ that includes η. Hence, unacceptable subsets are those for which there is insufficient predictive probability (under ℳ) that the out-of-sample accuracy of 𝒮 is within a predetermined margin of the best subset. The acceptable family is nonempty, since ${𝒮}_{min}\in A_{\eta ,\varepsilon }$, and is expanded by increasing η or decreasing ε. By default, we select η=0% and ε=0.10 and assess robustness in the simulation study (see also Kowal, 2021; Kowal et al., 2021).

Within the acceptable family, we isolate two subsets of particular interest: the best subset ${𝒮}_{\text{min}}$ from (18) and the smallest acceptable subset,

(20) $${𝒮}_{\text{small}}≔\text{arg}\underset{𝒮\in A_{\eta ,\varepsilon }}{\text{min}}\left|𝒮\right|.$$

When ${𝒮}_{\text{small}}$ is nonunique, so that multiple subsets of the same minimal size are acceptable, we select from those subsets the one with the smallest empirical loss $L_{𝒮}^{out}$. By definition, ${𝒮}_{\text{small}}$ is the smallest set of covariates that satisfies the near-optimality condition in (19). As noted by Hastie et al. (2009) and others, selection based on minimum cross-validated error, such as ${𝒮}_{min}$, often produces models or subsets that are more complex than needed for adequate predictive accuracy. The acceptable family $A_{\eta ,\varepsilon }$, and in particular ${𝒮}_{\text{small}}$, exploits this observation to provide alternative—and often much smaller—subsets of variables.

To ease the computational burden, we adapt the importance sampling algorithm from Kowal (2021) to compute (19) and all constituent quantities (see Appendix A). Crucially, this algorithm is based entirely on the in-sample posterior distribution under model ℳ, which avoids both (i) the intensive process of re-fitting ℳ for each data split k and (ii) data reuse that adversely affects downstream uncertainty quantification. Briefly, the algorithm uses the complete data posterior $p_{ℳ}\left(\theta |y\right)$ as a proposal for the training data posterior $p_{ℳ}\left(\theta |y^{-{ℐ}_{k}}\right)$. The importance weights are then computed using the likelihood $p_{ℳ}\left(y^{{ℐ}_{k}}|\theta \right)$ under model ℳ. Similar algorithms have been deployed for Bayesian model selection (Gelfand et al., 1992), evaluating prediction distributions (Vehtari and Ojanen, 2012), and $\ell_{1}$-based Bayesian variable selection (Kowal et al., 2021).

In the case of new (out-of-sample) covariate values ${\left\{{\overset{˜}{x}}_{i}\right\}}_{i=1}^{\tilde{n}}\neq {\left\{x_{i}\right\}}_{i=1}^{n}$, the predictive loss may be defined without cross-validation: ${\tilde{L}}_{𝒮}^{out}≔{\tilde{n}}^{-1}\sum_{i=1}^{\tilde{n}}L\left({\tilde{y}}_{i},{\overset{˜}{x}}_{i}^{\prime }{\overset{ˆ}{\delta }}_{𝒮}\right)$, where ${\tilde{y}}_{i}≔\overset{˜}{y}\left({\overset{˜}{x}}_{i}\right)$ is the predictive variable at ${\tilde{x}}_{i}$ for each $i=1,\ldots ,\tilde{n}$. The empirical loss is undefined without a corresponding observation of the response variable for each ${\overset{˜}{x}}_{i}$. Hence, we modify the acceptable family (19) to instead reference the full subset 𝒮={1,…,p} in place of ${𝒮}_{min}$, which is no longer defined. When ℳ is a linear model, Corollary 2 implies that the corresponding reference is simply the posterior predictive expectation under ℳ.

### 2.5. Co-variable importance

Although it is common to focus on a single subset for selection, the acceptable family $A_{\eta ,\varepsilon }$ provides a broad assortment of competing explanations: linear actions with distinct sets of covariates that all provide near-optimal predictive accuracy. Hence, we seek to further summarize $A_{\eta ,\varepsilon }$ beyond ${𝒮}_{min}$ and ${𝒮}_{\text{small}}$ to identify (i) which covariates appear in any acceptable subset, (ii) which covariates appear in all acceptable subsets, and (iii) which covariates appear together in the same acceptable subsets.

For covariates j and ℓ, the sample proportion of joint inclusion in $A_{\eta ,\varepsilon }$ achieves each of these goals:

(21) $${\text{VI}}_{\text{incl}}\left(j,\ell \right)≔{\left|A_{\eta ,\varepsilon }\right|}^{-1}\underset{𝒮\in A_{\eta ,\varepsilon }}{\sum }I\left\{j,\ell \in 𝒮\right\}$$

and measures *(co-) variable importance*. Naturally, (21) is generalizable to more than two covariates, but is particularly interesting for a single covariate: ${\text{VI}}_{\text{incl}}\left(j\right)≔{\text{VI}}_{\text{incl }}\left(j,j\right)$ is the proportion of acceptable subsets to which covariate j belongs. When ${\text{VI}}_{\text{incl }}\left(j\right)>0$, covariate j belongs to at least one acceptable subset. Such a result does not imply that covariate j is necessary for accurate prediction, but rather that covariate j is a component of at least one near-optimal linear subset. When ${\text{VI}}_{\text{incl }}\left(j\right)=1$, we refer to covariate j as a *keystone covariate*: it belongs to all acceptable subsets and therefore is deemed essential. For $j\neq \ell ,{\text{VI}}_{\text{incl }}\left(j,\ell \right)$ highlights not only the covariates that co-occur, but also the covariates that rarely appear together. It is particularly informative to identify covariates j and ℓ such that ${\text{VI}}_{\text{incl}}\left(j\right)$ and ${\text{VI}}_{\text{incl}}\left(\ell \right)$ are both large yet ${\text{VI}}_{\text{incl}}\left(j,\ell \right)$ is small. In that case, covariates j and ℓ are both important yet also redundant, as might be expected for highly correlated variables.

Although ${\text{VI}}_{\text{incl }}$ is defined based on linear predictors for each subset 𝒮, the variable importance metric applies broadly to (possibly nonlinear) Bayesian models ℳ and a variety of evaluative loss functions L, and can be targeted locally via the weights $\omega \left({\overset{˜}{x}}_{i}\right)$. The inclusion-based metric ${\text{VI}}_{\text{incl }}$ can be extended to incorporate effect size, which is a more common strategy for variable importance. Related, Dong and Rudin (2019) aggregated model-specific variable importances across many “good models”. Alternative approaches use leave-one-covariate-out predictive metrics (e.g., Lei et al., 2018), but are less appealing in the presence of correlated covariates.

### 2.6. Posterior predictive uncertainty quantification

A persistent challenge in classical subset selection is the lack of accompanying uncertainty quantification. Given a subset $\hat{𝒮}$ selected using the data (X,y), familiar frequentist and Bayesian inferential procedures applied to $\left(X_{\hat{𝒮}},y\right)$ are in general no longer valid. In particular, we cannot simply proceed as if only the selected covariates $\hat{𝒮}$ were supplied from the onset. Such analyses are subject to selection bias (Miller, 1984).

A crucial feature of our subset filtering (Section 2.2 and Section 2.3) and predictive evaluation (Section 2.4) techniques is that, despite the broad searching and the out-of-sample targets, these quantities all remain *in-sample posterior functionals* under ℳ. There is no data re-use or model re-fitting: every requisite term is a functional of the complete data posterior $p_{ℳ}\left(\theta |y\right)$ or $p_{ℳ}\left(\overset{˜}{y}|y\right)$ from a single Bayesian model. Hence, the posterior distribution under ℳ remains a valid facilitator of uncertainty quantification.

We elicit a posterior predictive distribution for the action by removing the expectation in (2):

(22) $$\overset{˜}{\delta }≔\text{arg }\underset{\delta }{\text{min }}ℒ\left(\overset{˜}{y},\delta \right)$$

which no longer integrates over $p_{ℳ}\left(\overset{˜}{y}|y\right)$ and hence propagates the posterior predictive uncertainty. For the squared error loss (3), the *predictive action* is

(23) $${\tilde{\delta }}_{𝒮}={\left({\tilde{X}}_{𝒮}^{\prime }\Omega {\tilde{X}}_{𝒮}\right)}^{-1}{\tilde{X}}_{𝒮}^{\prime }\Omega \tilde{y}$$

akin to (5), where $\overset{˜}{y}={\left({\tilde{y}}_{1},\ldots ,{\tilde{y}}_{\tilde{n}}\right)}^{\prime }\sim p_{ℳ}\left(\overset{˜}{y}|y\right)$. The linear coefficients ${\overset{˜}{\delta }}_{𝒮}$ inherit a posterior predictive distribution from $\overset{˜}{y}$ and can be computed for any subset 𝒮. Similar computations are available for the cross-entropy loss (10). In both cases, draws ${\left\{{\overset{˜}{y}}^{s}\right\}}_{s=1}^{S}\sim p_{ℳ}\left(\overset{˜}{y}|y\right)$ from the posterior predictive distribution are sufficient for uncertainty quantification of ${\overset{˜}{\delta }}_{𝒮}$. Under the usual assumption that $p_{ℳ}\left(\overset{˜}{y}|\theta ,y\right)=p_{ℳ}\left(\overset{˜}{y}|\theta \right)$, these draws are easily obtained by repeatedly sampling $\theta^{s}\sim p_{ℳ}\left(\theta |y\right)$ from the posterior and ${\overset{˜}{y}}^{s}\sim p_{ℳ}\left(\overset{˜}{y}|\theta =\theta^{s}\right)$ from the likelihood.

The predictive actions are computable for any subset 𝒮, include those selected based on the predictive evaluations in Section 2.4. Let $\hat{𝒮}$ denote a subset identified based on the posterior (predictive) distribution under ℳ, such as ${𝒮}_{min}$ or ${𝒮}_{\text{small}}$. The predictive action ${\overset{˜}{\delta }}_{\hat{𝒮}}$ using this subset in (23) is a posterior predictive functional. Unlike for a generic subset 𝒮, the predictive action ${\overset{˜}{\delta }}_{\hat{𝒮}}$ is a functional of both $\overset{˜}{y}$ and $\hat{𝒮}$, which factors into the interpretation.

A similar projection-based approach is developed by Woody et al. (2020) for Gaussian regression. In place of $ℒ\left(\overset{˜}{y},\delta \right)$ in (22), Woody et al. (2020) suggest $E_{\overset{˜}{y}|\theta }ℒ\left(\overset{˜}{y},\delta \right)$, which is a middle ground between (2) and (22): it integrates over the uncertainty of $\overset{˜}{y}$ given model parameters θ, but preserves uncertainty due to θ. For example, under the linear regression model $E_{\overset{˜}{y}|\theta }\left\{\overset{˜}{y}\left(\overset{˜}{x}\right)\right\}={\overset{˜}{x}}^{\prime }\beta$, the analogous result in (23) is ${\left({\overset{˜}{X}}_{𝒮}^{\prime }\Omega {\overset{˜}{X}}_{𝒮}\right)}^{-1}{\overset{˜}{X}}_{𝒮}^{\prime }\Omega \overset{˜}{X}\beta$; when 𝒮={1,…,p}, this simplifies to the regression coefficient β. Both approaches have their merits, but we prefer (22) because of the connection to the observable random variables $\overset{˜}{y}$ rather than the model-specific parameters θ.

## 3. Simulation study

We conduct an extensive simulation study to evaluate competing subset selection techniques for prediction accuracy, uncertainty quantification, and variable selection. Prediction for real-valued data is discussed here; classification for binary data is presented in Appendix B. Although we do evaluate individual subsets from the proposed framework—namely, ${𝒮}_{min}$ and ${𝒮}_{\text{small}}$—we are more broadly interested in the performance of the *acceptable family* of subsets. In particular, the acceptable family is designed to collect *many subsets* that offer *near-optimal* prediction; both cardinality and aggregate predictive accuracy are critical.

The simulation designs feature varying signal-to-noise ratios (SNRs) and dimensions (n,p), including p≫n, with correlated covariates and sparse linear signals. Covariates $x_{i,j}$ are generated from marginal standard normal distributions with $\text{Cor}\left(x_{i,j},x_{i,j\prime }\right)={\left(0.75\right)}^{\left|j-j\prime \right|}$ for i=1,…,n and j=1,…,p. The p columns are randomly permuted and augmented with an intercept. The true linear coefficients $\beta^{\text{*}}$ are constructed by setting $\beta_{0}^{\text{*}}=-1$ and fixing $p_{\text{*}}=5$ nonzero coefficients, with $\left\lceil p_{\text{*}}/2\right\rceil$ equal to 1 and $\left\lfloor p_{\text{*}}/2\right\rfloor$ equal to −1, and the rest at zero. The data are generated independently as $y_{i}\sim N\left(y_{i}^{\text{*}},\sigma_{\text{*}}^{2}\right)$ with $y_{i}^{\text{*}}≔x_{i}^{\prime }\beta^{\text{*}}$ and $\sigma_{\text{*}}≔\text{sd}\left(y^{\text{*}}\right)/\sqrt{\text{SNR}}$. We consider (n,p)∈{(50,50),(200,400),(500,50)} and SNR∈{0.25,1} for low and high SNR. Evaluations are conducted over 100 simulated datasets.

First, we evaluate point prediction accuracy across competing collections of near-optimal subsets. Each collection is built from the same Gaussian linear regression model with horseshoe priors (Carvalho et al., 2010) ℳ and estimated using bayeslm (Hahn et al., 2019). The collections are generated from different candidate sets S based on distinct search methods: the proposed BBA method (bbound(bayes)), the adaptive lasso search (lasso (bayes)) proposed by Hahn and Carvalho (2015) and Kowal et al. (2021), and both forward (forward(bayes)) and backward (backward(bayes)) search. For each collection of candidate subsets, we compute the acceptable families for $\eta =0\text{\%},\varepsilon =0.10,m_{k}=15$, and the observed covariate values ${\left\{{\overset{˜}{x}}_{i}\right\}}_{i=1}^{\tilde{n}}={\left\{x_{i}\right\}}_{i=1}^{n}$; variations of these specifications are presented in Appendix C. These methods differ *only* in the search process: if a particular subset 𝒮 is identified by more than one of these competing methods, the corresponding point predictions—based on the optimal action ${\overset{ˆ}{\delta }}_{𝒮}$ in (5)—will be identical.

**Figure 1:**
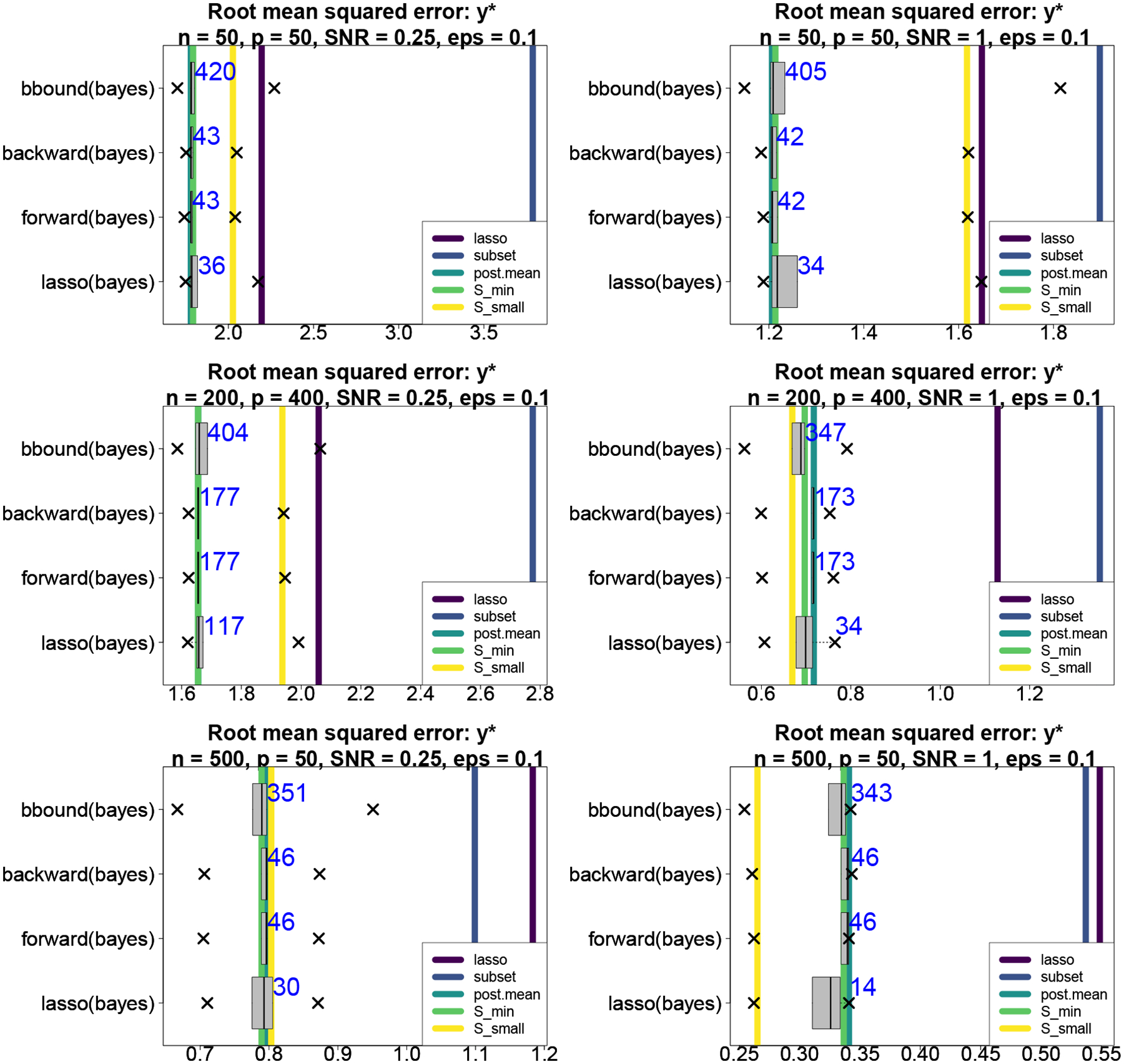
Root mean squared errors (RMSEs) for predicting $y^{\text{*}}$ across (n,p,SNR) configurations. The boxplots summarize the RMSE quantiles for the subsets within each acceptable family, while the vertical lines denote RMSEs of competing methods. The average size of each acceptable family is annotated. The proposed BBA search returns vastly more subsets that remain highly competitive, while ${𝒮}_{\text{small}}$ performs very well and substantially outperforms classical subset selection.

The competing collections of near-optimal subsets are evaluated in aggregate. At each simulation, we compute the *q*th quantile of the root mean squared errors (RMSEs) for the true mean ${\left\{y_{i}^{\text{*}}\right\}}_{i=1}^{n}$ across all subsets within that acceptable family, and then average that quantity across all simulations. For example, q=1 describes the predictive performance if we were to use the worst acceptable subset at each simulation, as determined by an oracle. These quantities summarize the distribution of RMSEs *within* each acceptable family; we report the values for q∈{0,0.25,0.5,0.75,1} and present the results as boxplots in Figure 1. Most notably, the proposed bbound(bayes) strategy produces up to 10 times the number of acceptable subsets as the other methods, yet crucially maintains equivalent predictive performance. Naturally, this expanded collection of subsets also produces a minimum RMSE (q=0) that outperforms the remaining methods. Clearly, the proposed approach is far superior at collecting *more* subsets that provide *near-optimal* predictive accuracy.

Figure 1 also summarizes the point prediction accuracy for several competing methods. First, we include the usual point estimate under ℳ given by the posterior expectation of the regression coefficients β (post.mean), which does not include any variable or subset selection. Next, we compute point predictions for the key acceptable subsets ${𝒮}_{min}$ and ${𝒮}_{\text{small}}$ using the proposed bbound(bayes) approach; the competing search strategies produced similar results and are omitted. Among frequentist estimators, we use the adaptive lasso (lasso; Zou, 2006) with λ chosen by 10-fold cross-validation and the one-standard-error rule (Hastie et al., 2009). In addition, we include classical subset selection (subset) implemented using the leaps package in R with the final subset determined by AIC. When p>35, we screen to the first 35 predictors that enter the model in the aforementioned adaptive lasso. Forward (forward) and backward (backward) selection were also considered, but were either noncompetitive or performed similarly to the adaptive lasso and are omitted from this plot.

Most notably, the predictive performance of ${𝒮}_{\text{small}}$ is excellent, especially for high SNRs, and substantially outperforms the frequentist methods in all settings. Because ${𝒮}_{min}$ is overly conservative—i.e., it selects too many variables (see Table 1)—it performs nearly identically to post.mean. Although ${𝒮}_{min}$ and post.mean outperform ${𝒮}_{\text{small}}$ when the SNR is low and the sample size is small, note that ${𝒮}_{\text{small}}$ is not targeted exclusively for optimal predictive performance; rather, it represents the smallest subset that obtains *near-optimal* performance. Lastly, the regularization induced by ℳ is greatly beneficial: *every* member of each acceptable family decisively outperforms classical subset selection across all scenarios.

**Figure 2:**
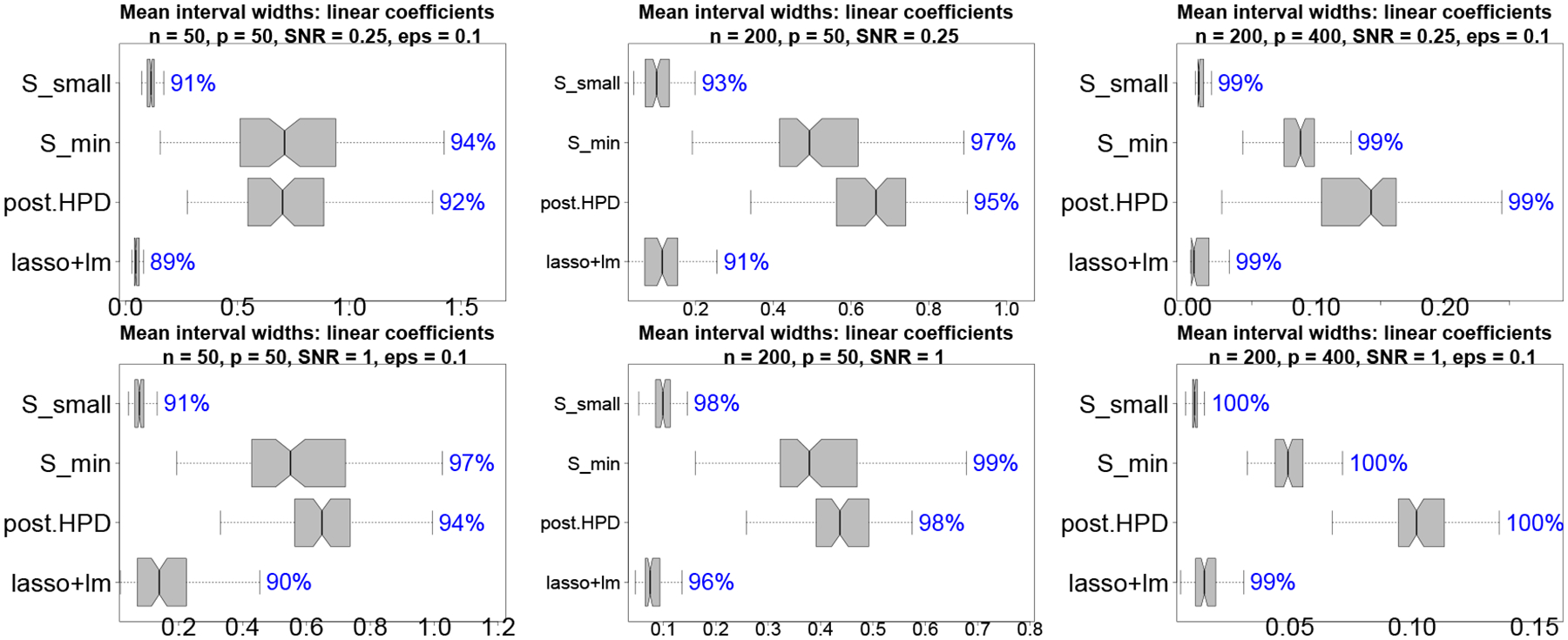
Mean interval widths (boxplots) with empirical coverage (annotations) for $\beta^{\text{*}}$. Non-overlapping notches indicate significant differences between medians. The proposed intervals based on ${𝒮}_{\text{small}}$ are significantly narrower than the usual HPD intervals under ℳ yet maintain the empirical nominal 90% coverage.

Next, we compare uncertainty quantification for the regression coefficients $\beta^{\text{*}}$. We compute 90% intervals using the predictive action ${\overset{˜}{\delta }}_{𝒮}$ from (23) for ${𝒮}_{\text{small}}$ and ${𝒮}_{min}$ based on the proposed bbound(bayes) procedure. In addition, we compute 90% highest posterior density (HPD) credible intervals for β under ℳ (post.HPD) and 90% frequentist confidence intervals using Zhao et al. (2021), which computes confidence intervals from a linear regression model that includes only the variables selected by the (adaptive) lasso (lasso+lm). The 90% interval estimates are evaluated and compared in Figure 2 using interval widths and empirical coverage. The intervals from ${𝒮}_{\text{small}}$ using (22) are uniformly better (i.e., narrower) than the usual posterior HPD intervals under ℳ. In addition, the intervals from Zhao et al. (2021) are highly competitive, despite ignoring selection bias. In all cases, the intervals are overly conservative and achieve the nominal empirical coverage.

Lastly, marginal variable selection is evaluated using true positive rates and true negative rates in Table 1. In addition to ${𝒮}_{min}$ and ${𝒮}_{\text{small}}$, we include the common Bayesian selection technique that includes a variable j when the 95% HPD interval for $\beta_{j}$ excludes zero (posterior HPD). The selection performance of ${𝒮}_{\text{small}}$ is excellent and similar to the the adaptive lasso. Classical subset, forward, and backward selection are too aggressive, while the posterior interval-based selection is too conservative. Hence, despite the primary focus on the *collection* of near-optimal subsets, the smallest acceptable subset ${𝒮}_{\text{small}}$ is a key member with excellent prediction, uncertainty quantification, and marginal selection performance.

The appendix contains simulation studies for classification (Appendix B) and variations for $\varepsilon \in \left\{0.01,0.10,0.20\right\},{\left\{{\overset{˜}{x}}_{i}\right\}}_{i=1}^{\tilde{n}}$, and the distributions of ${\left\{x_{i}\right\}}_{i=1}^{n}$ and ${\left\{{\overset{˜}{x}}_{i}\right\}}_{i=1}^{\tilde{n}}$ (Appendix C). All results are qualitatively similar to those presented here.

## 4. Subset selection for predicting educational outcomes

**Figure 3:**
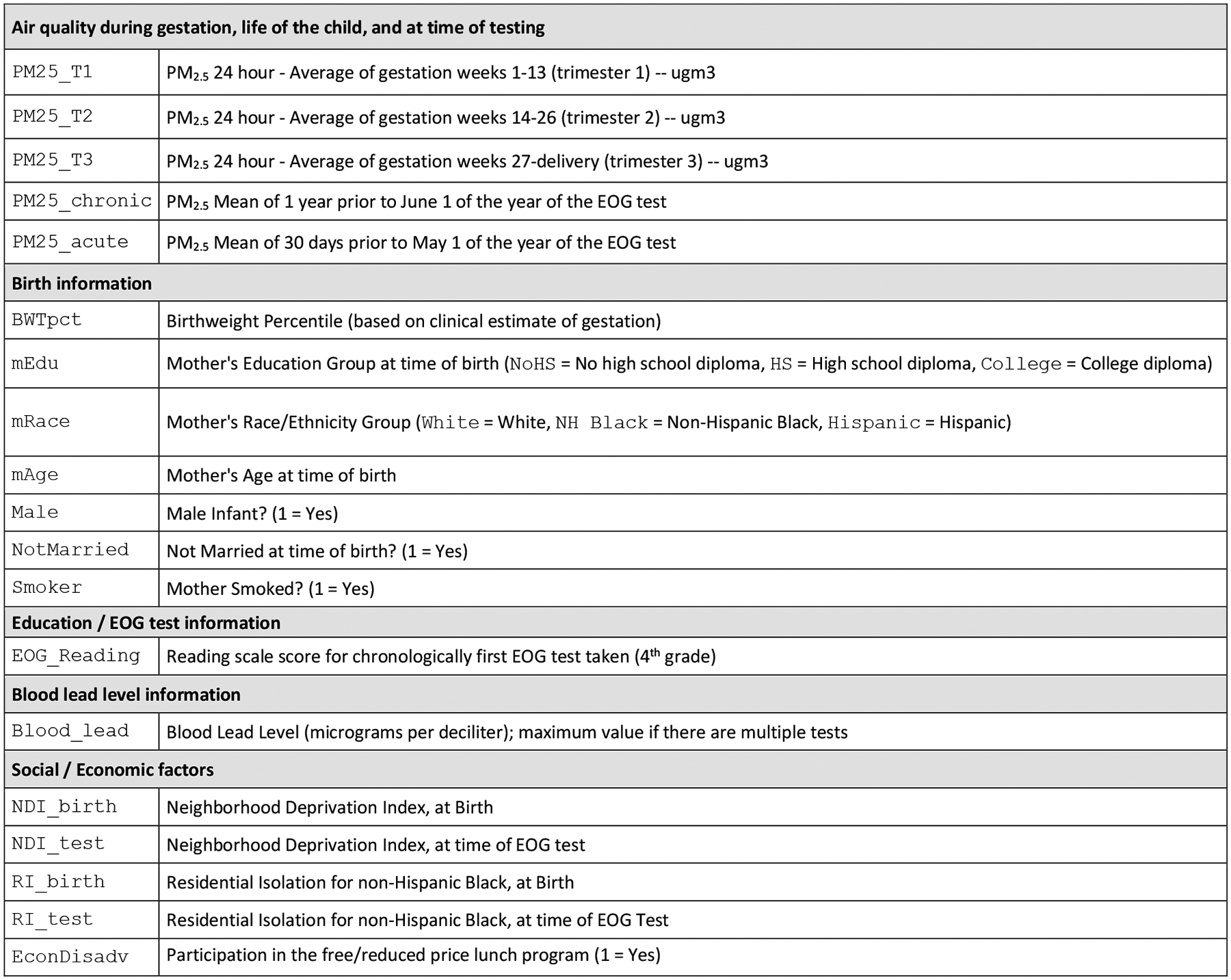
Variables in the NC education dataset. Data are restricted to individuals with 37–42 weeks gestation, mother’s age 15–44, Blood_lead ≤80, birth order ≤4, no current limited English proficiency, and residence in NC at time of birth and time of 4th grade test.

Childhood educational outcomes are affected by adverse environmental exposures, such as poor air quality and lead, as well as social stressors, such as poverty, racial residential isolation, and neighborhood deviation. We study childhood educational development using end-of-grade reading test scores for a large cohort of fourth grade children in North Carolina (Children’s Environmental Health Initiative, 2020). The reading scores are accompanied by a collection of student-level information detailed in Figure 3, which includes air quality exposures, birth information, blood lead measurements, and social and economic factors (see also Kowal et al., 2021). The goal is to identify subsets of these variables and interactions that offer near-optimal prediction of reading scores as well as accurate classification of at-risk students.

**Figure E.1:**
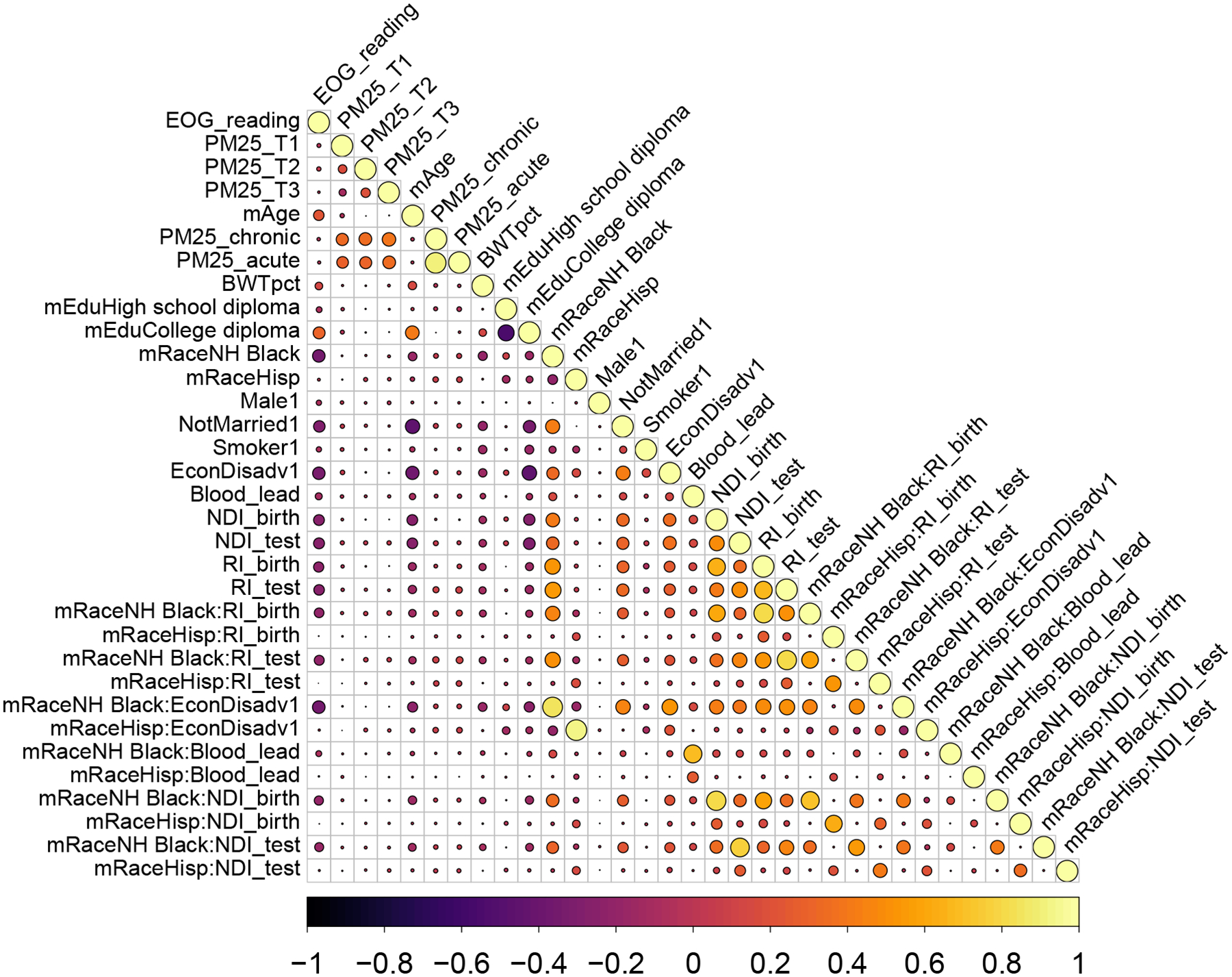
Pairwise correlations among the covariates in the NC education data.

A prominent feature of the data is the correlation among the covariates. After centering and scaling the continuous covariates to mean zero and standard deviation 0.5, we augment the variables in Figure 3 (excluding the test scores) with interactions between race and the social and economic factors. The resulting dimensions are n=16806 and p=32. Figure E.1 displays the pairwise correlations among the covariates and the response variable. There are strong associations among the air quality exposures as well as among race and the social and economic factors. Due to the dependences among variables, it is likely that distinct subsets of similar predictive ability can be obtained by interchanging among these correlated covariates. Hence, it is advantageous to collect and study the near-optimal subsets.

We compute acceptable families for (i) the reading scores $y_{i}$ under squared error loss and (ii) the indicator $h\left({\tilde{y}}_{i}\right)=I\left\{{\tilde{y}}_{i}\geq \tau_{0.1}\right\}$ under cross-entropy loss, where $\tau_{0.1}$ is the 0.1-quantile of the reading scores (see Appendix D). While task (i) broadly considers the spectrum of educational outcomes via reading scores, task (ii) targets at-risk students in the bottom 10% of reading ability. Acceptable families and accompanying quantities for both tasks can be computed using the same Bayesian model ℳ : we focus on Gaussian linear regression with horseshoe priors. The acceptable families are computed using the proposed BBA search with $\eta =0\text{\%},\varepsilon =0.10,m_{k}=100$ and ${\left\{{\overset{˜}{x}}_{i}\right\}}_{i=1}^{\tilde{n}}={\left\{x_{i}\right\}}_{i=1}^{n}$; results for other η values, ε=0.20, and $m_{k}=15$ are noted, while alternative target covariates ${\left\{{\overset{˜}{x}}_{i}\right\}}_{i=1}^{\tilde{n}}$ are in Section 4.2.

### 4.1 Subset selection for predicting reading scores

**Figure 4:**
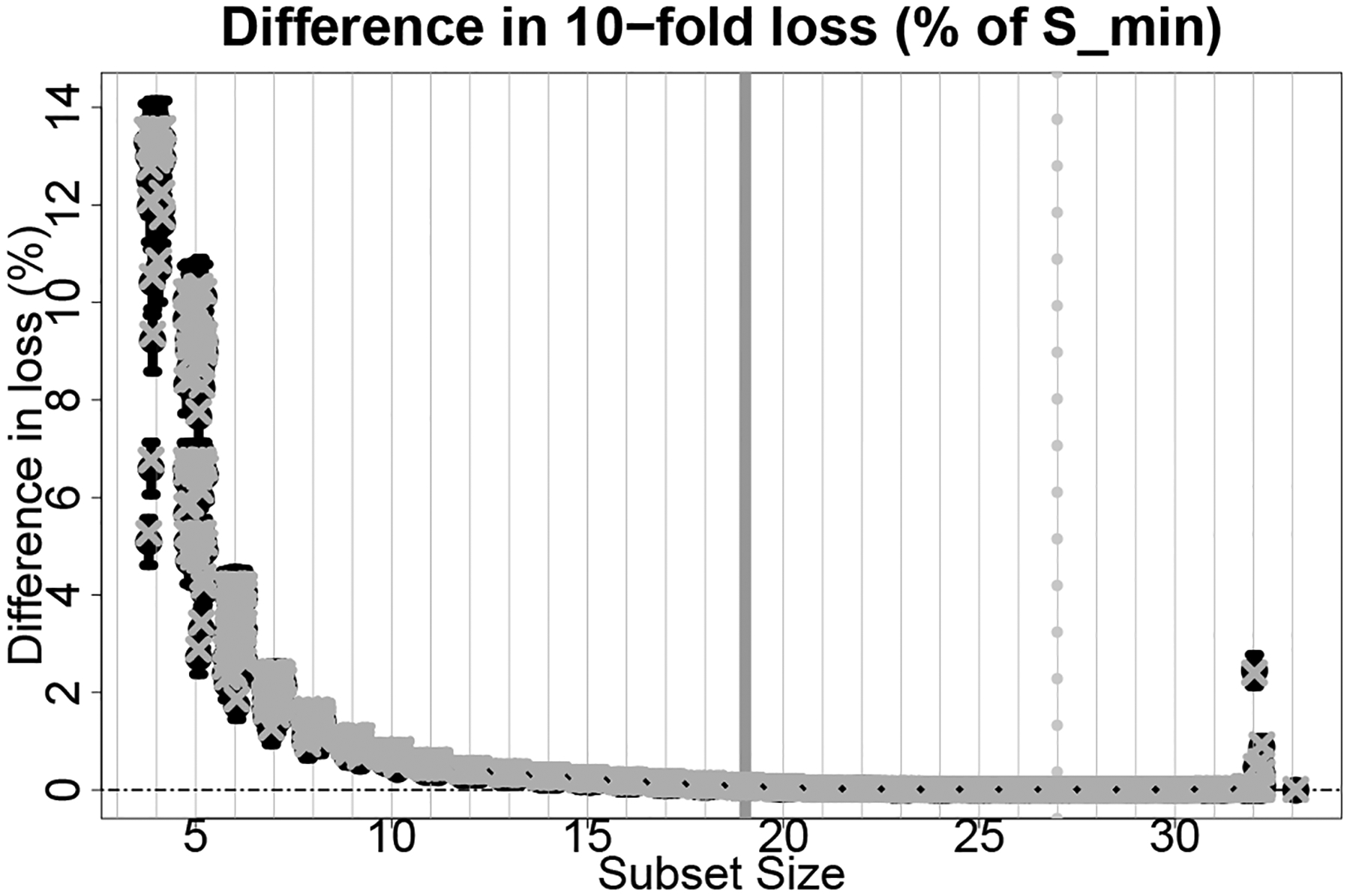
The 80% intervals (bars) and expected values (circles) for ${\tilde{D}}_{{𝒮}_{min},𝒮}^{out}$ with ${\tilde{D}}_{𝒮,{𝒮}_{min}}^{out}$ (x-marks) under squared error loss for each subset size |𝒮| with 𝒮∈S. We annotate ${𝒮}_{min}$ (dashed gray line) and ${𝒮}_{\text{small}}$ (solid gray line) for ε=0.10 and η=0 and jitter the subset sizes for clarity of presentation.

First, we predict reading scores using a linear model for ℳ and squared error loss for L. Since acceptable family is defined based on ${\tilde{D}}_{{𝒮}_{min},𝒮}^{out}$ in (19), we summarize its distribution in Figure 4. For each 𝒮∈S, we display 80% intervals, expectations, and the empirical analog $D_{𝒮,{𝒮}_{min}}^{out}≔100\times \left(L_{𝒮}^{out}-L_{{𝒮}_{min}}^{out}\right)/L_{{𝒮}_{min}}^{out}$. The smaller subsets of sizes four to six demonstrate clear separation for certain subsets. Along with the intercept and the race indicators, these subsets include mEdu (college diploma), EconDisadv, and mEdu (completed high school) in sequence. However, larger subsets are needed to procure near-optimal predictions for smaller margins, such as η<2%. While the best subset $\left|{𝒮}_{min}\right|=29$ includes nearly all of the covariates, many subsets with |𝒮|>10 achieve within η=1% of the accuracy of ${𝒮}_{\text{min }}$.

**Figure 5:**
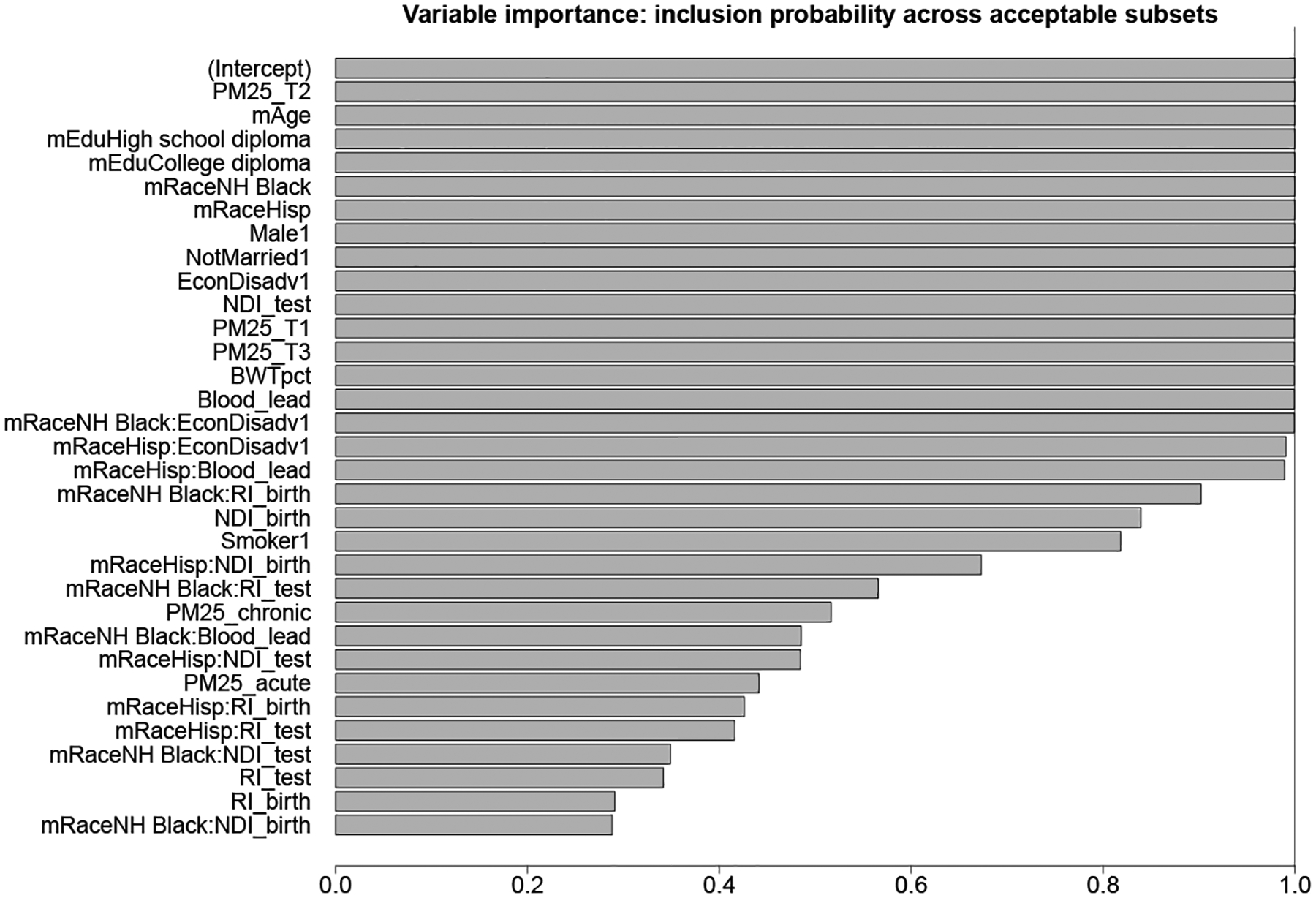
Variable importance ${\text{VI}}_{\text{incl }}\left(j\right)$ for prediction. There are several tiers: variables appear in (nearly) all, many (> 70%), some (> 30%), or no acceptable subsets.

Among the |S|=2761 candidate subsets identified from the BBA search, there are $\left|A_{0,0.1}\right|=1183$ acceptable subsets. We summarize $A_{\eta ,\varepsilon }$ via the co-variable importance metrics ${\text{VI}}_{\text{incl}}\left(j\right)$ and ${\text{VI}}_{\text{incl}}\left(j,\ell \right)$ in Figures 5 and E.2, respectively. Unlike many variable importance metrics that measure effect sizes, ${\text{VI}}_{\text{incl }}\left(j\right)$ instead quantifies whether each covariate j is a component of all, some, or no competitive subsets. There are many keystone covariates that appear in (nearly) all acceptable subsets, including environmental exposures (prenatal air quality and blood lead levels), economic and social factors (EconDisadv, mother’s education level, neighborhood deprivation at time of test), and demographic information (race, gender), among others.

**Figure E.2:**
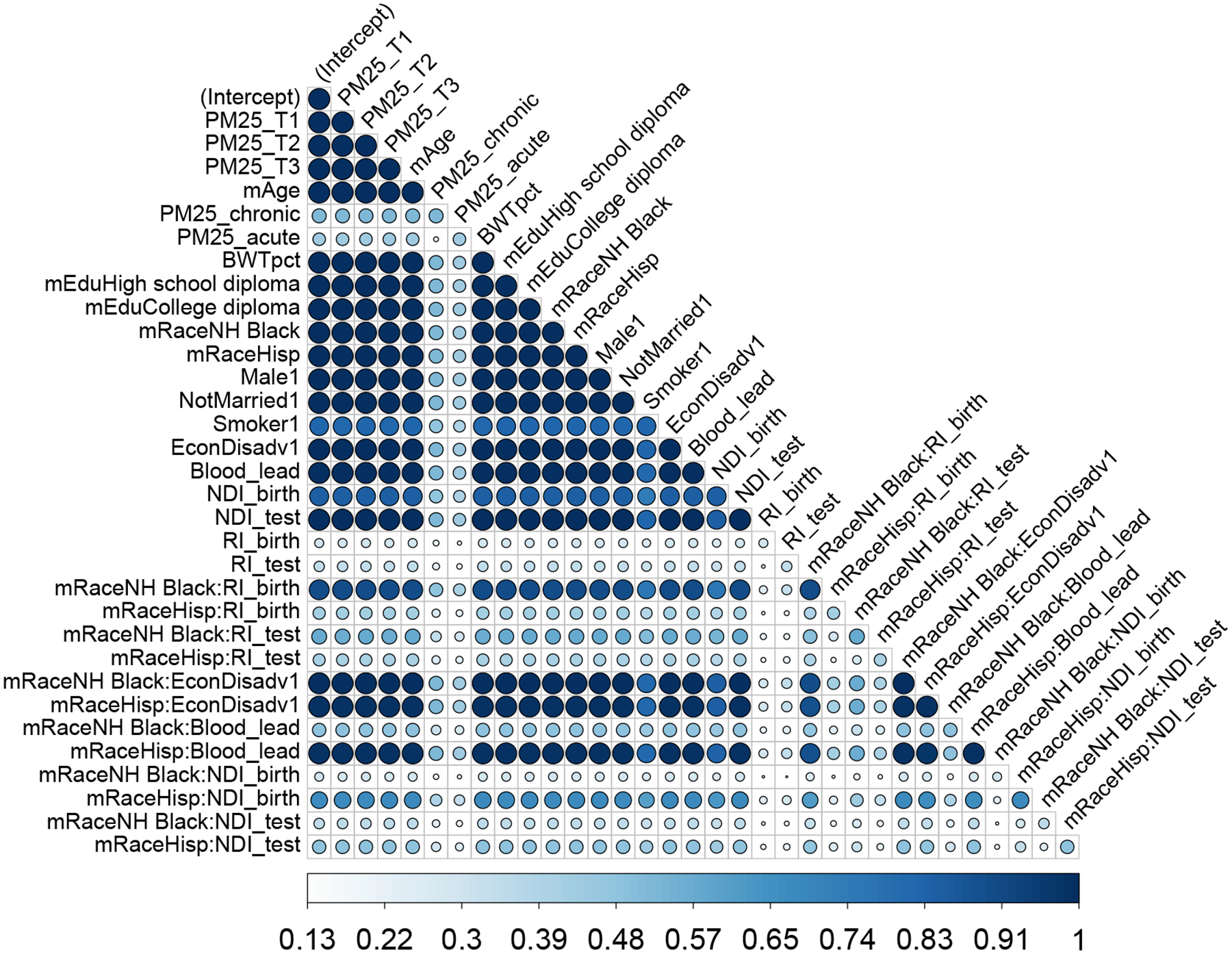
Co-variable importance ${\text{VI}}_{\text{incl}}\left(j,\ell \right)$ for prediction. For certain pairs of variables (chronic and acute PM_2.5_ exposure; neighborhood deprivation and racial residential isolation both at birth and time of test), it is common for one—but not both—to appear in an acceptable subset.

Interestingly, chronic and acute ${\text{PM}}_{2.5}$ exposure each belong to nearly 50% of acceptable subsets (Figure 5), yet rarely appear in the same acceptable subset (Figure E.2). The pairwise correlations (Figure E.1) offer a reasonable explanation: these variables are weakly correlated with reading scores but highly correlated with one another. Similar results persist for neighborhood deprivation and racial residential isolation both at birth and time of test. Moreover, this analysis was conducted after removing one acute ${\text{PM}}_{2.5}$ outlier (50% larger than all other values). When that outlier is kept in the data, acute ${\text{PM}}_{2.5}$ no longer belongs to any acceptable subset, while ${\text{VI}}_{\text{incl }}\left(j\right)$ for chronic ${\text{PM}}_{2.5}$ increases. These results are encouraging: the acceptable family identifies redundant yet distinct predictive explanations, but prefers the more stable covariate in the presence of outliers.

**Figure 6:**
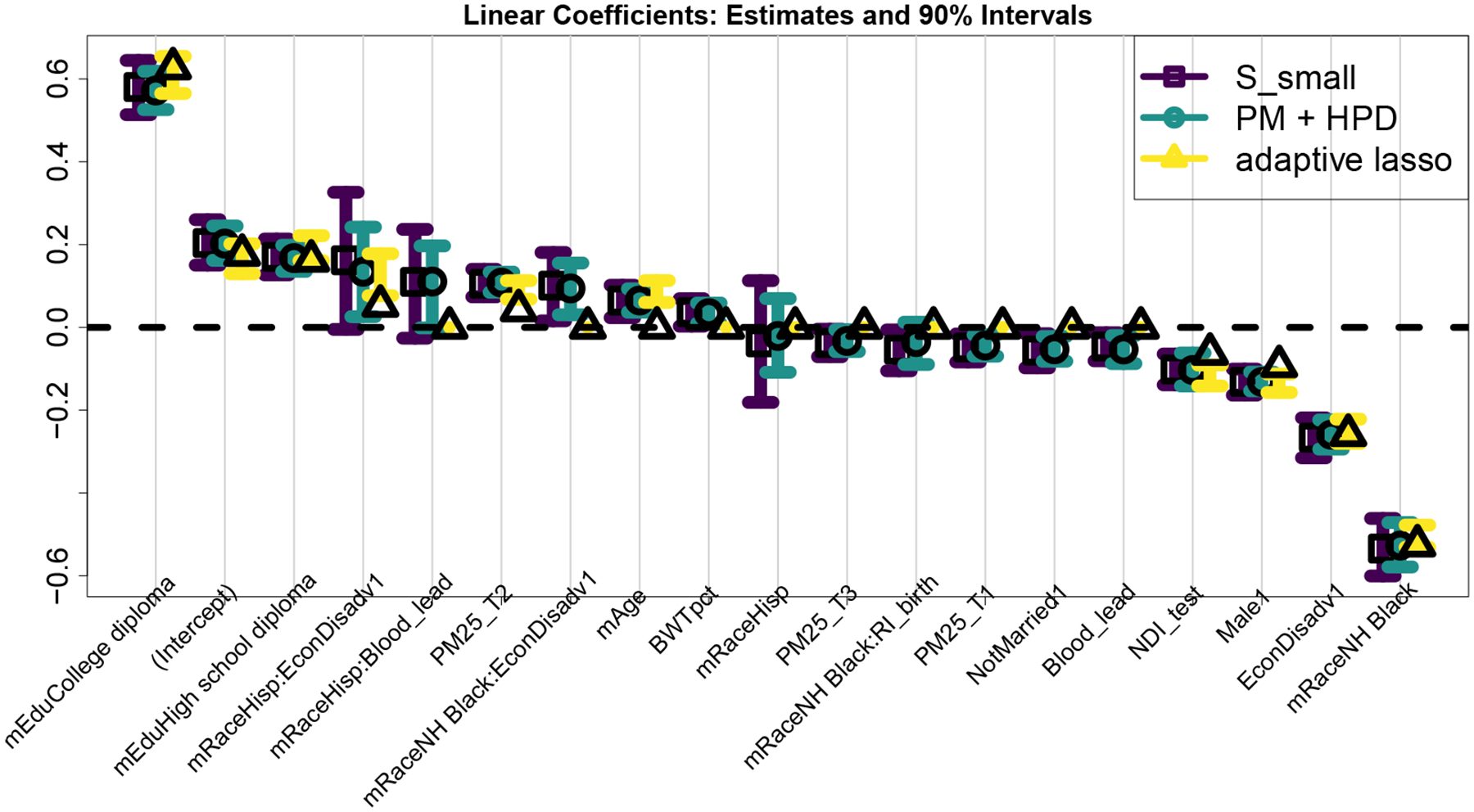
Estimated linear effects and 90% intervals for the variables in ${𝒮}_{\text{small}}$ based on the proposed approach, model ℳ, and the adaptive lasso.

Next, we analyze the smallest acceptable subset ${𝒮}_{\text{small}}$ and incorporate uncertainty quantification for the accompanying linear coefficients. The $\left|{𝒮}_{\text{small}}\right|=19$ selected covariates are displayed in Figure 6 alongside the point and 90% intervals based on the proposed approach, the Bayesian model ℳ, and the adaptive lasso. The estimates and intervals for covariates excluded from ${𝒮}_{\text{small}}$ are identically zero for the proposed approach (and, in this case, the adaptive lasso as well), while the estimates and HPD intervals from ℳ are dense for all covariates. Despite the encouraging simulation results for the Zhao et al. (2021) frequentist intervals, these intervals often exclude the adaptive lasso point estimates, which undermines interpretability.

The estimates from ${𝒮}_{\text{small}}$ and ℳ are similar with anticipated directionality: higher mother’s education levels, lower blood lead levels in the child, less neighborhood deprivation, and absence of economic disadvantages predict higher reading scores. Prenatal air quality exposure $\left({\text{PM}}_{2.5}\right)$ is significant: due to seasonal effects, the 1st and 3rd trimester exposures have negative coefficients, while the 2nd trimester has a positive effect. Naturally, these effects can only be interpreted jointly. Among interaction terms, the negative effect of NH Black × RI_birth suggests the lower reading scores among non-Hispanic Black students are accentuated by racial residential isolation in the neighborhood of the child’s birth. Since we do not force all main effects into each subset, ${𝒮}_{\text{small}}$ does not contain an estimated effect of RI_birth for other race groups. Other interactions, such as the positive effects of Hisp and NH Black by EconDisadv and Hisp × Blood_lead, must also be interpreted carefully: the vast majority of Hispanic and non-Hispanic Black students belong to the EconDisadv group and have much higher blood lead levels on average, while each of NH Black, EconDisadv, and Blood_lead has a strong negative main effect.

The results are not particularly sensitive to $m_{k}$ or ε. When $m_{k}=15$, there are |S|=436 candidate subsets and $\left|A_{0,0.1}\right|=197$ acceptable subsets. The variable importance metrics broadly agree with Figures 5 and E.2, while ${𝒮}_{\text{small}}$—and therefore Figure 6—is unchanged. When ε=0.20 (and $m_{k}=100$ as before), the acceptable family reduces slightly to $\left|A_{0,0.1}\right|=$ 977 members and ${𝒮}_{\text{small}}$ differs only in the addition of Smoker and Hisp × NDI_test.

### 4.2 Out-of-sample prediction

We evaluate the predictive capabilities of the proposed approach for 20 training/testing splits of the NC education data. The same competing methods are adopted from Section 3, including the distinct search strategies for collecting near-optimal subsets. Since ${𝒮}_{\text{small}}$ and ${𝒮}_{min}$ are reasonably robust to $m_{k}$, we select $m_{k}=15$ for computational efficiency. In addition, we include the acceptable family defined by setting ${\left\{{\overset{˜}{x}}_{i}\right\}}_{i=1}^{\tilde{n}}$ to be the testing data covariate values (bbound(Xtilde)), which is otherwise identical to bbound(bayes). Root mean squared prediction errors (RMSPEs) are used for evaluation.

**Figure 7:**
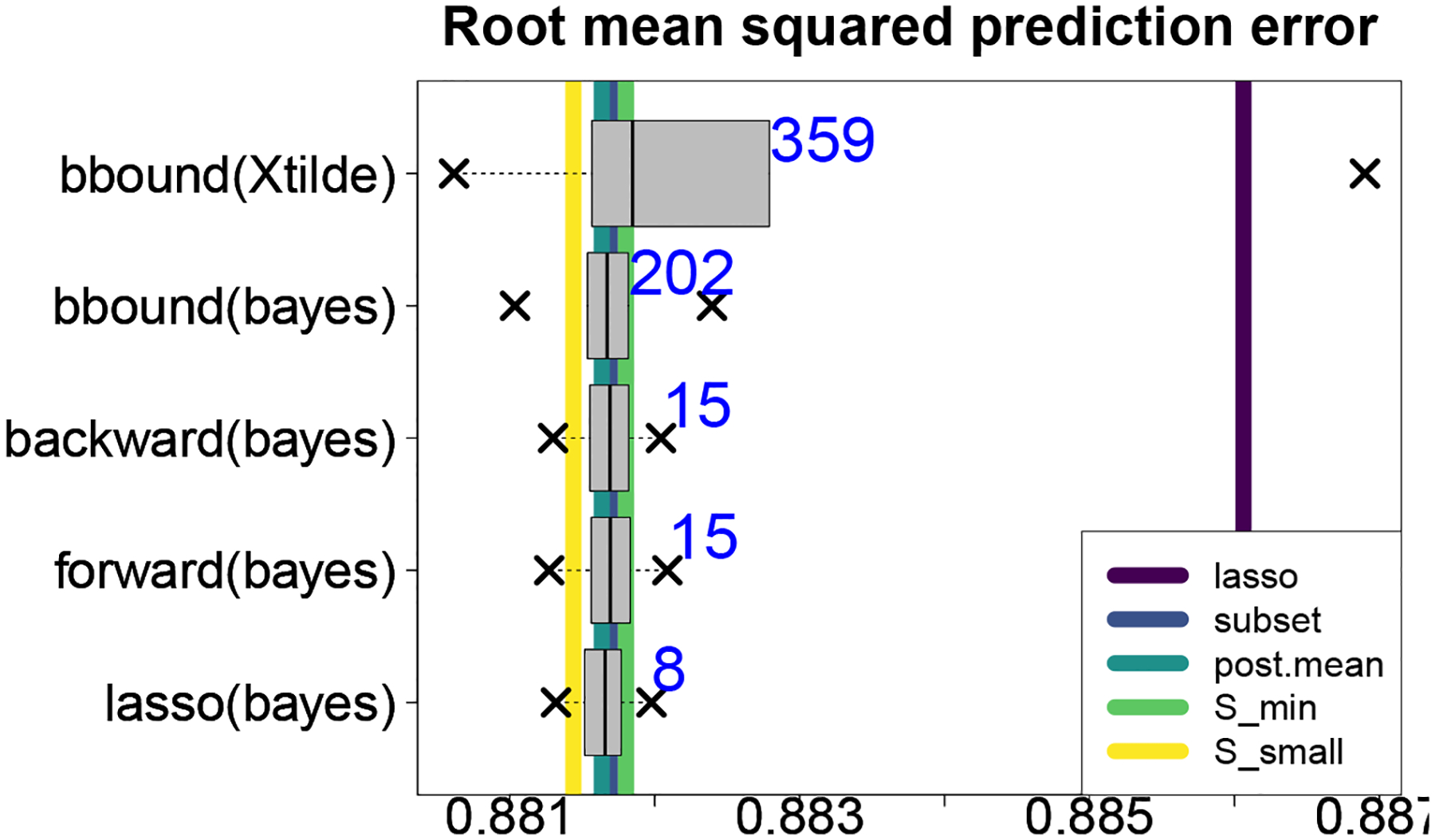
Root mean squared prediction errors (RMSPEs) across 20 training/testing splits of the NC education data. The boxplots summarize the RMSPE quantiles for the subsets within each acceptable family, while the vertical lines denote RMSPEs of competing methods. The average size of each acceptable family is annotated. The proposed BBA search returns vastly more subsets that remain highly competitive, while the accompanying ${𝒮}_{\text{small}}$ subset performs best overall.

The results are presented in Figure 7. Among single subset methods, ${𝒮}_{\text{small}}$ outperforms all competitors—including the classical forward and backward estimators and the smallest acceptable subsets from lasso(bayes), forward(bayes), and backward (bayes) discussed in Section 3 (not shown). The adaptive lasso selects fewer variables and is not competitive. Among search methods, Figure 7 confirms the results from the simulation study: the proposed BBA strategy (bbound (bayes)) identifies 10–25 times the number of subsets as the other search strategies, yet does not sacrifice any predictive accuracy in this expanded collection. Clearly, bbound(bayes) provides a more complete predictive picture, as the competing search strategies omit a massive number of subsets that *do* offer near-optimal prediction. The acceptable family based on the out-of-sample covariates bbound(Xtilde) is much larger, which is reasonable: the subsets are computed and evaluated on covariates for which the accompanying response variables are not available. This collection of subsets sacrifices some predictive accuracy relative to the other search strategies, yet still outperforms the adaptive lasso—yet another testament to the importance of the regularization induced by ℳ.

Although the RMSPE differences appear to be small, minor improvements are practically relevant: even a single point on a standardized test score can be the difference between progression to the next grade level (on the low end) or eligibility for intellectually gifted programs (on the high end). More generally, education data are prone to weak signals and small effect sizes, which are conditions under which *many* methods may offer similar predictive performance. Nonetheless, our primary goal is not to substantially *improve* prediction, but rather to identify and analyze a large collection of near-optimal subsets.

The importance of the broader BBA search strategy is further highlighted in comparison lasso(bayes), which uses a lasso search path for decision analytic Bayesian variable selection (Hahn and Carvalho, 2015; Kowal et al., 2021). In particular, lasso(bayes) generates only |S|=25 candidate subsets and $\left|A_{0,0.1}\right|=9$ acceptable subsets. By comparison, bbound(bayes) returns more than 100 times the number of candidate subsets *and* acceptable subsets. Figure 7 shows that the subsets omitted by lasso(bayes) yet discovered by bbound(bayes) are indeed near-optimal. Further, the restrictive search path of lasso(bayes) does not guarantee greater stability: the smallest acceptable subsets for bbound(bayes) and lasso(bayes) are nearly identical, yet the interquartile range of $\left|{𝒮}_{\text{small}}\right|$ across the 20 training/testing splits under bbound(bayes) is 0, while the same quantity under lasso(bayes) is 2. Indeed, by this metric, bbound(bayes) actually provides the *most stable* smallest acceptable subset across all search strategies considered.

## 5. Discussion

We developed decision analysis tools for Bayesian subset search, selection, and (co-) variable importance. The proposed strategy is outlined in Algorithm 1. Building from a Bayesian predictive model ℳ, we derived optimal linear actions for any subset of covariates. We explored the space of subsets using an adaptation of the branch-and-bound algorithm. After filtering to a manageable collection of promising subsets, we identified the *acceptable family* of near-optimal subsets for linear prediction or classification. The acceptable family was summarized by a new (co-) variable importance metric—the frequency with which variables (co-) appear in all, some, or no acceptable subsets—and individual member subsets, including the “best” and smallest subsets. Using the posterior predictive distribution from ℳ, we developed point and interval estimates for the linear coefficients of any subset. Simulation studies demonstrated better prediction, interval estimation, and variable selection for the proposed approach compared to existing Bayesian and frequentist selection methods—even for high-dimensional data with p>n.

We applied these tools to a large education dataset to study the factors that predict educational outcomes. The analysis identified several keystone covariates that appeared in (almost) every near-optimal subset, including environmental exposures, economic and social factors, and demographic information. The co-variable importance metrics highlighted an interesting phenomenon where certain pairs of covariates belonged to many acceptable subsets, yet rarely appeared in the same acceptable subset. Hence, these variables are effectively interchangeable for prediction, which provides valuable context for interpreting their respective effects. We showed that the smallest acceptable subset offers excellent prediction of end-of-grade reading scores and classification of at-risk students using substantially fewer covariates. The corresponding linear coefficients described new and important effects, for example that greater racial residential isolation among non-Hispanic Black students is predictive of lower reading scores. However, our results also caution against overreliance on any particular subset: we identified over 200 distinct subsets of variables that offer near-optimal out-of-sample predictive accuracy.

Future work will attempt to generalize these tools via the loss functions and the actions. Alternatives to squared error and cross-entropy loss can be incorporated with an IRLS approximation strategy similar to Section 2.3, which would maintain the methodology and algorithmic infrastructure from the proposed approach. Similarly, the class of parametrized actions can be expanded to include nonlinear predictors, such as trees or additive models, with acceptable families constructed in the same way.


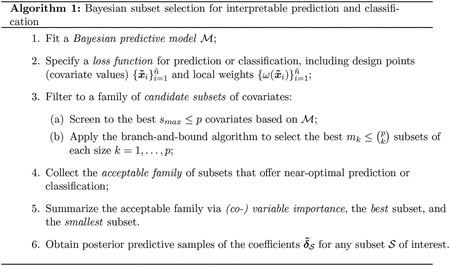


## Figures and Tables

**Table 1:** True positive rates (TPR) and true negative rates (TNR) for Gaussian synthetic data with $p^{\text{*}}+1=6$ active covariates including the intercept. The selection performance of ${𝒮}_{\text{small}}$ is excellent and similar to the the adaptive lasso. Classical subset, forward, and backward selection are too aggressive, while the posterior interval-based selection is too conservative.

n=50, p=50, SNR = 0.25							
	lasso	forward	backward	subset	posterior HPD	$S_{min}$	$S_{\text{small}}$
TPR	0.22	0.96	0.93	0.53	0.06	0.51	0.22
TNR	0.98	0.06	0.10	0.63	1.00	0.81	0.98
n=50, p=50, SNR = 1							
	lasso	forward	backward	subset	posterior HPD	$S_{min}$	$S_{\text{small}}$
TPR	0.54	0.95	0.89	0.72	0.16	0.77	0.34
TNR	0.92	0.06	0.11	0.64	1.00	0.78	0.99
n=200, p=400, SNR = 0.25							
	lasso	forward	backward	subset	posterior HPD	$S_{min}$	$S_{\text{small}}$
TPR	0.36	0.79	0.78	0.67	0.17	0.75	0.34
TNR	1.00	0.52	0.54	0.96	1.00	0.95	1.00
n=200, p=400, SNR = 1							
	lasso	forward	backward	subset	posterior HPD	$S_{min}$	$S_{\text{small}}$
TPR	0.97	0.96	0.95	0.95	0.75	0.98	0.93
TNR	0.99	0.57	0.60	0.96	1.00	0.95	1.00
n=500, p=50, SNR = 0.25							
	lasso	forward	backward	subset	posterior HPD	$S_{min}$	$S_{\text{small}}$
TPR	0.84	0.96	0.95	0.94	0.59	0.99	0.86
TNR	0.98	0.83	0.83	0.83	1.00	0.70	0.98
n=500, p=50, SNR = 1							
	lasso	forward	backward	subset	posterior HPD	$S_{min}$	$S_{\text{small}}$
TPR	1.00	1.00	1.00	1.00	1.00	1.00	1.00
TNR	1.00	0.84	0.83	0.83	1.00	0.67	0.99

**Figure B.1:**
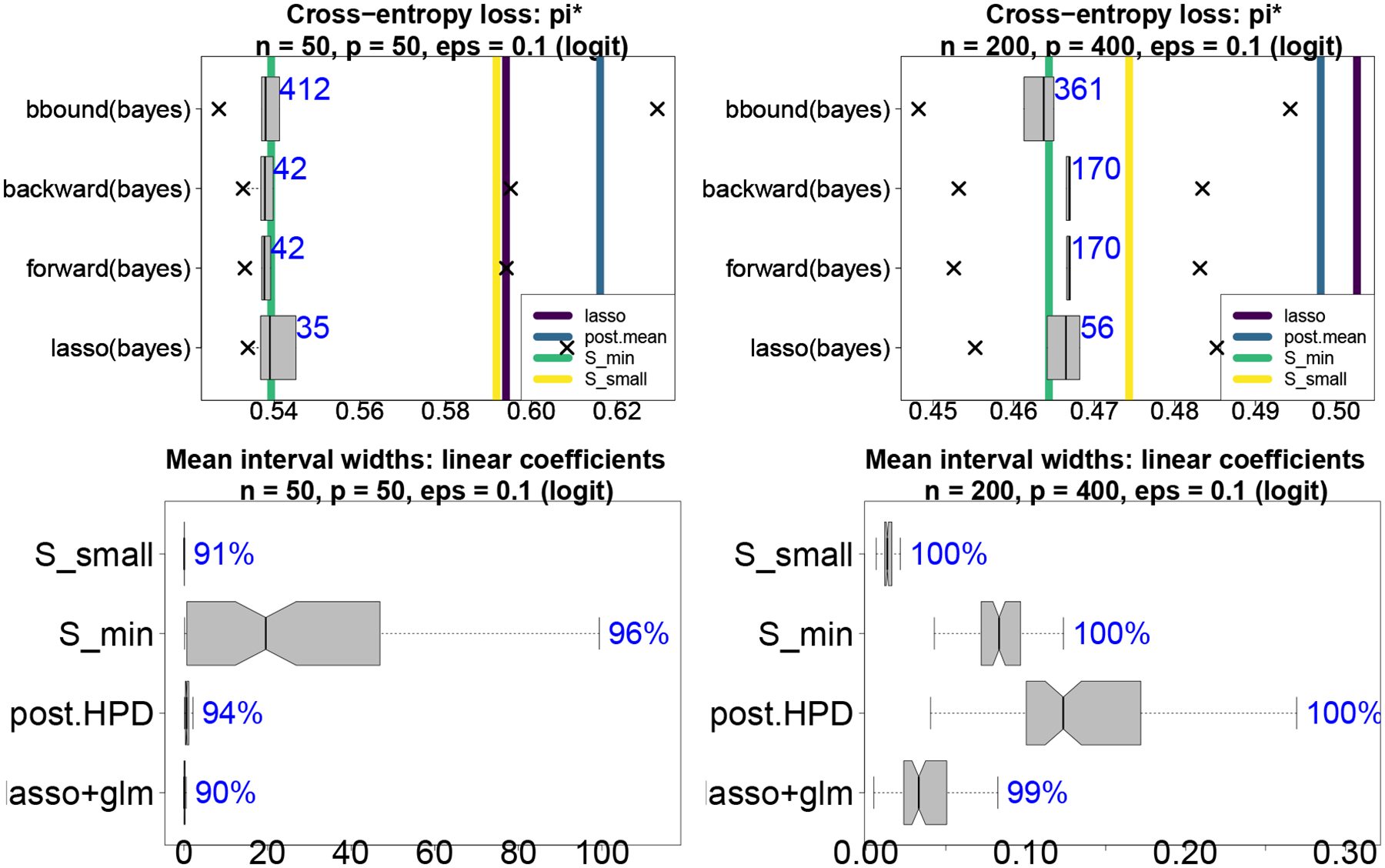
**Top:** cross-entropy loss for $\pi_{i}^{\text{*}}$. The boxplots summarize the cross-entropy quantiles for the subsets within each acceptable family, while the vertical lines denote cross-entropy of competing methods. The average size of each acceptable family is annotated. **Bottom:** Mean interval widths (boxplots) with empirical coverage (annotations) for $\beta^{\text{*}}$.

**Figure C.1:**
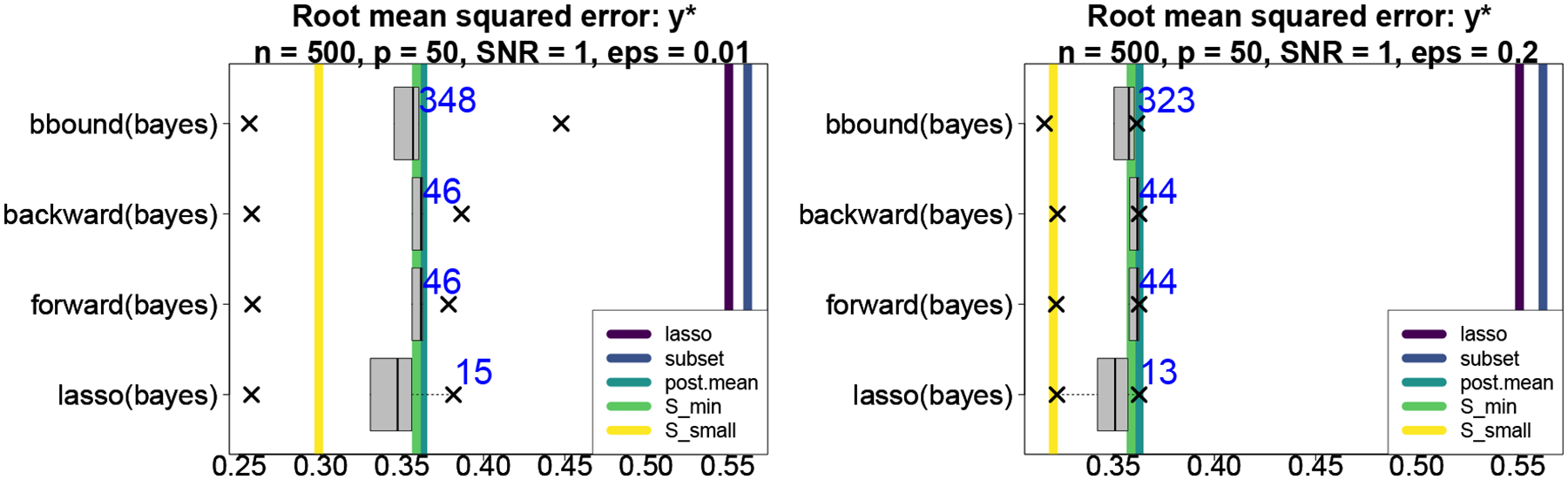
Root mean squared errors (RMSEs) for predicting $y^{\text{*}}$. The boxplots summarize the RMSE quantiles for the subsets within each acceptable family; here, the acceptable families use ε=0.01 (left) and ε=0.20 (right). The vertical lines denote RMSEs of competing methods and the average size of each acceptable family is annotated. The results suggest only minor sensitivity to the choice of ε.

**Figure C.2:**
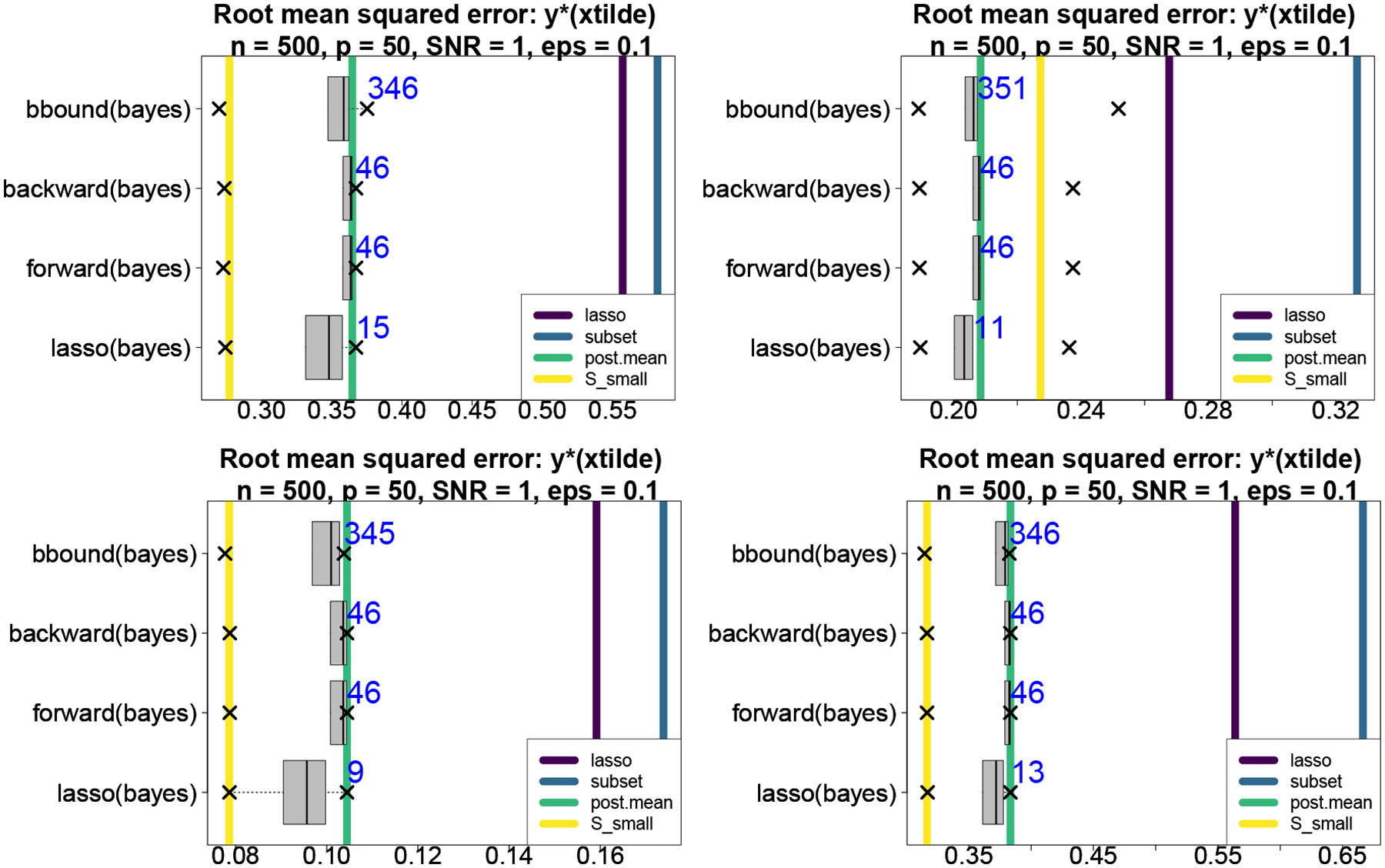
Root mean squared errors (RMSEs) for predicting $y^{\text{*}}\left({\overset{˜}{x}}_{i}\right)={\overset{˜}{x}}_{i}^{\prime }\beta^{\text{*}}$ for covariates ${\left\{{\overset{˜}{x}}_{i}\right\}}_{i=1}^{\tilde{n}=1000}$ when the observed covariates ${\left\{x_{i}\right\}}_{i=1}^{n}$ are correlated standard normals (top) or iid uniforms (bottom) and when the target covariates ${\left\{{\overset{˜}{x}}_{i}\right\}}_{i=1}^{\tilde{n}=1000}$ have the same distribution (left) or a different distribution (right) from ${\left\{x_{i}\right\}}_{i=1}^{n}$. The boxplots summarize the RMSE quantiles for the subsets within each acceptable family. The vertical lines denote RMSEs of competing methods and the average size of each acceptable family is annotated.

**Figure D.1:**
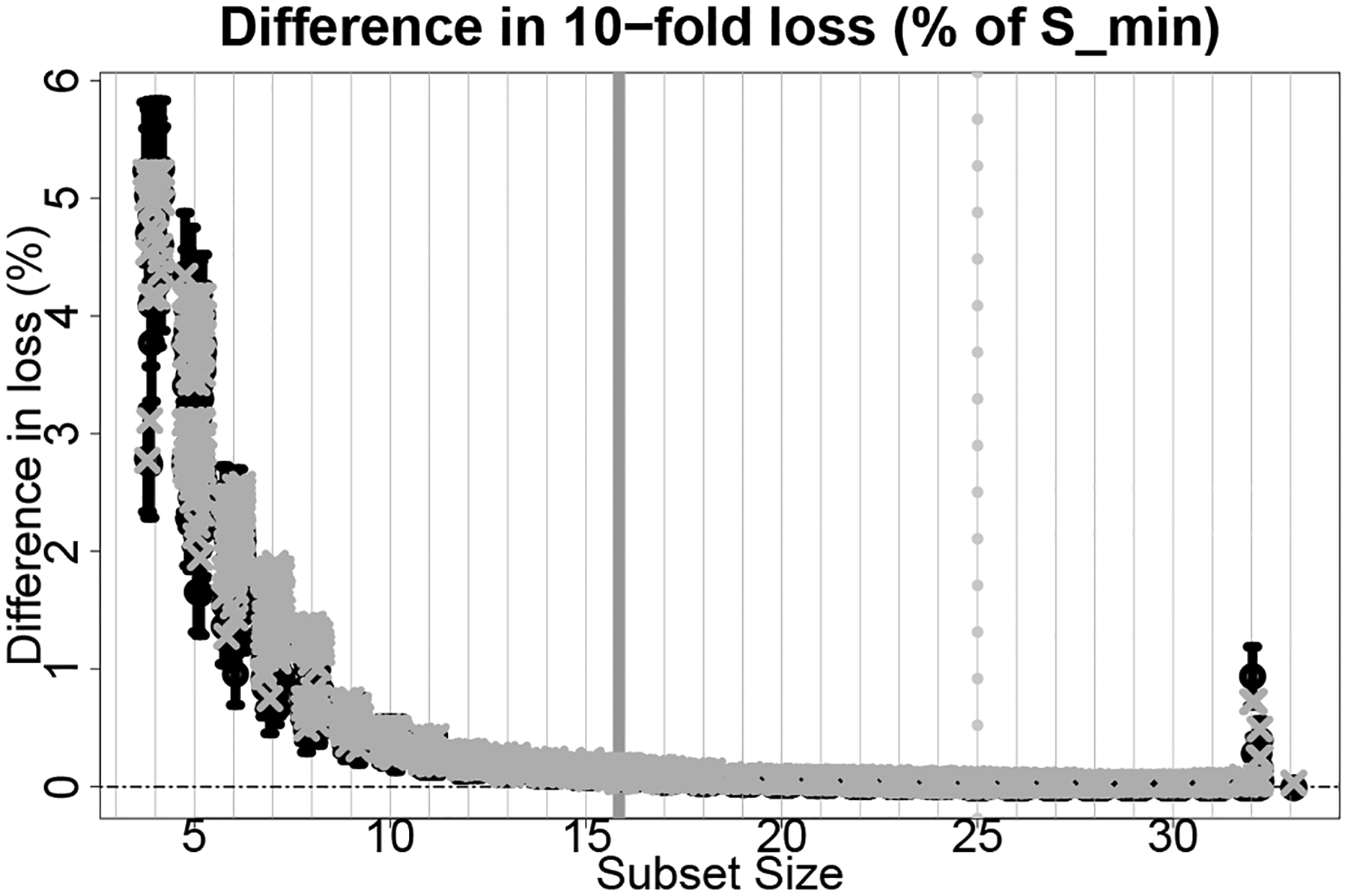
The 80% intervals (bars) and expected values (circles) for ${\tilde{D}}_{{𝒮}_{min},𝒮}^{out}$ with $D_{𝒮,{𝒮}_{min}}^{out}$ (x-marks) under cross-entropy for each subset size |𝒮| with 𝒮∈S. We annotate ${𝒮}_{\text{min}}$ (dashed gray line) and ${𝒮}_{\text{small}}$ (solid gray line) and jitter the subset sizes for clarity of presentation.

**Figure D.2:**
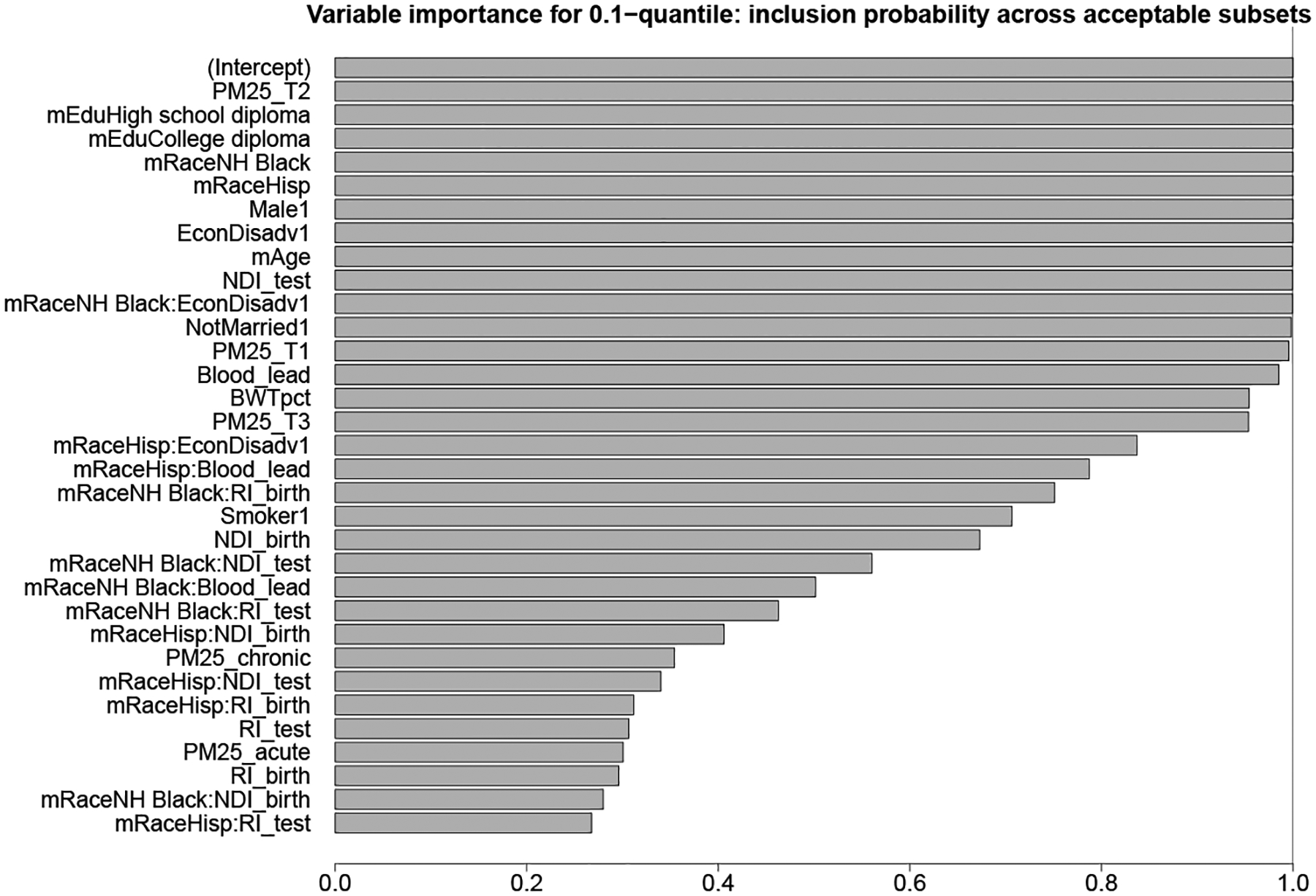
Variable importance ${\text{VI}}_{\text{incl}}\left(j\right)$ for classification of the 0.1-quantile $\left(m_{k}=100\right)$.

**Figure D.3:**
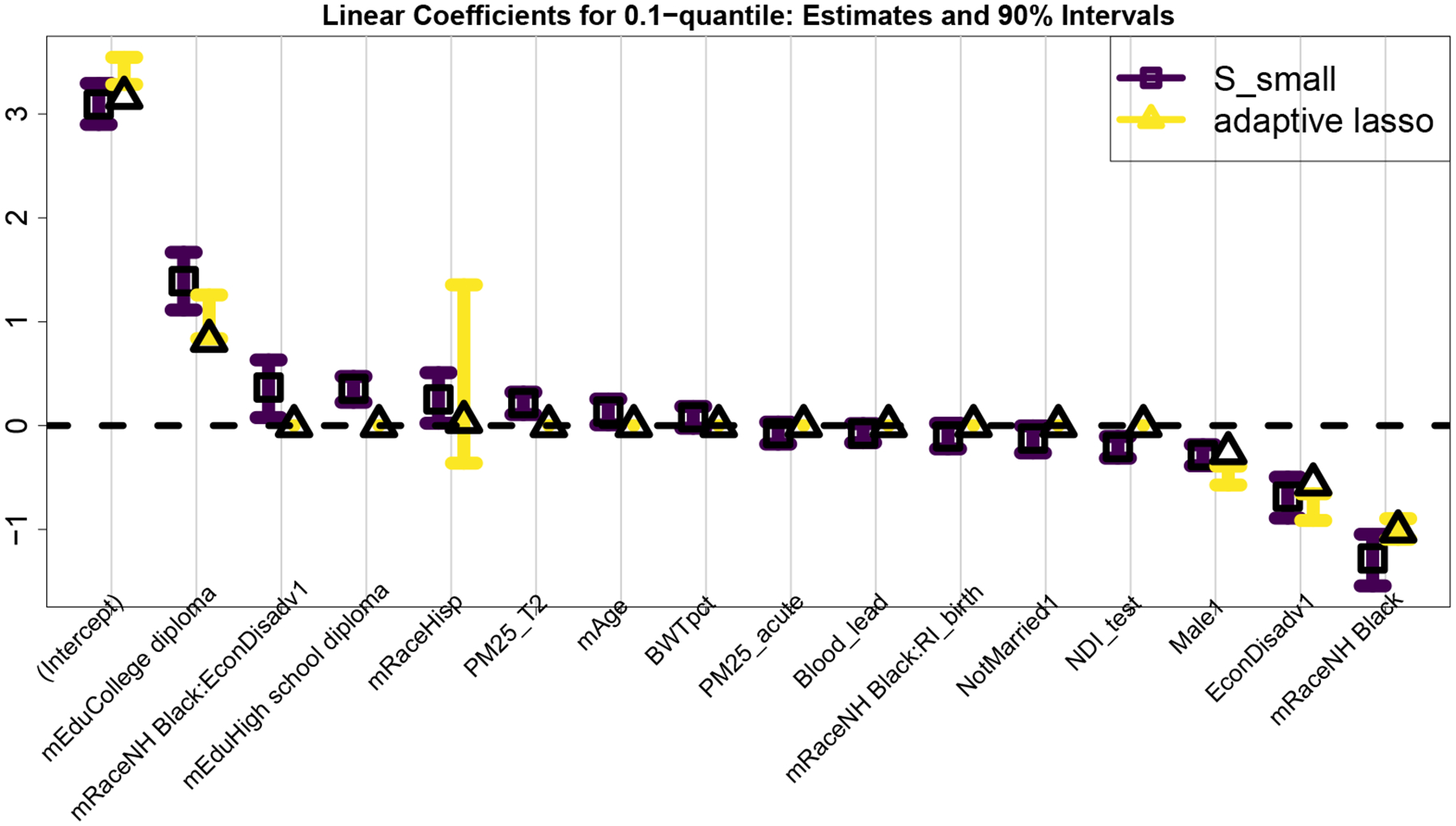
Estimated linear effects and 90% intervals for the variables in ${𝒮}_{\text{small}}$ based on the proposed approach and the adaptive lasso.

**Figure E.3:**
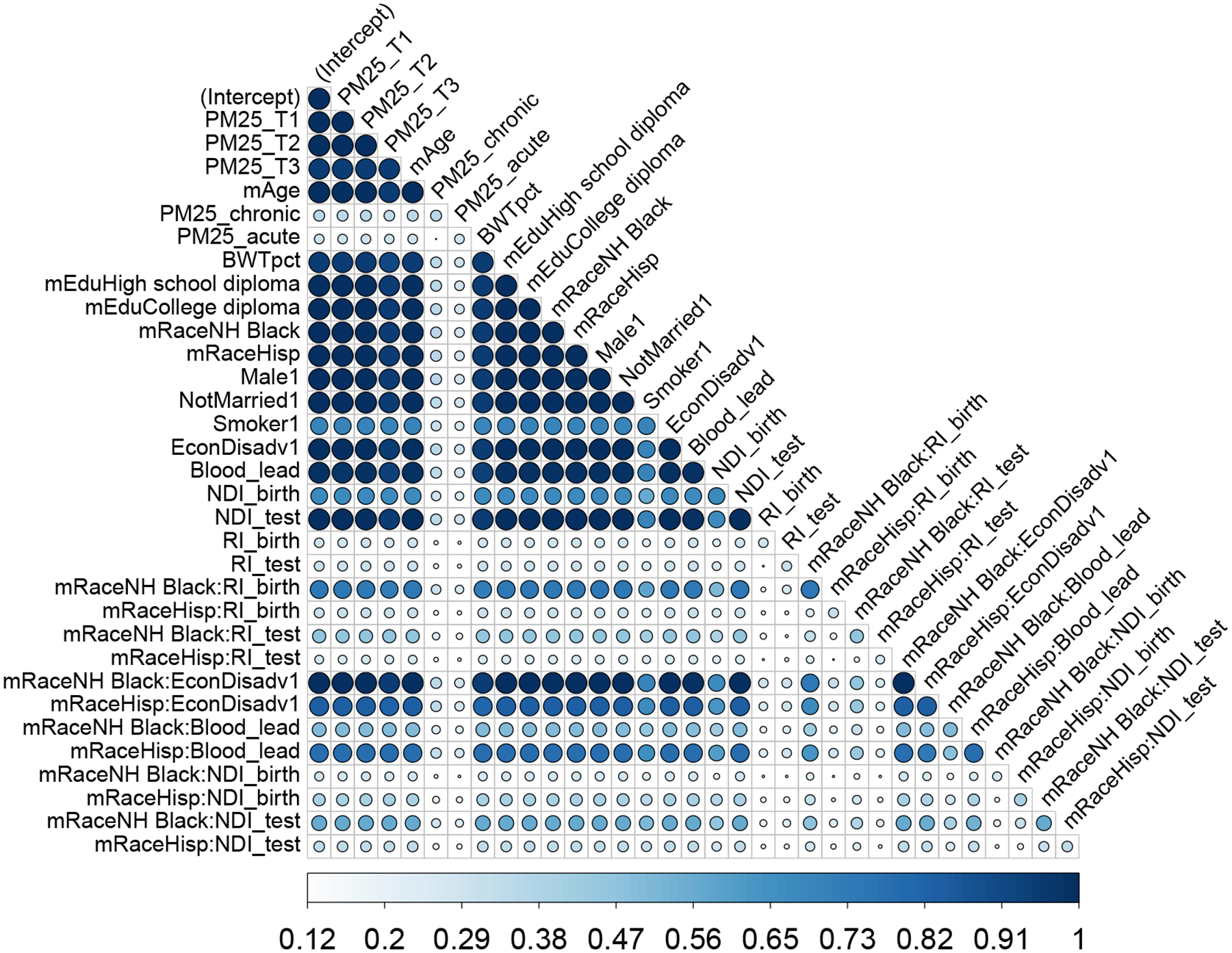
Co-variable importance ${\text{VI}}_{\text{incl}}\left(j,\ell \right)$ for classification of the 0.1-quantile.

## Appendix A. Approximations for out-of-sample quantities

The acceptable family $A_{\eta ,\varepsilon }$ in (19) is based on the out-of-sample predictive discrepancy metric ${\tilde{D}}_{{𝒮}_{1},{𝒮}_{2}}^{out}$ and the best subset ${\overset{ˆ}{\delta }}_{{𝒮}_{min}}$ in (18). These quantities both depend on the out-of-sample empirical and predictive losses $L_{𝒮}^{out}\left(k\right)$ and ${\tilde{L}}_{𝒮}^{out}\left(k\right)$, respectively, in (16). Hence, it is required to compute (i) the optimal action on the training data, ${\overset{ˆ}{\delta }}_{𝒮}^{-{ℐ}_{k}}≔{\text{arg min}}_{\delta_{𝒮}}E_{\overset{˜}{y}|y^{-{ℐ}_{k}}}ℒ\left({\left\{{\tilde{y}}_{i}\right\}}_{i\in {ℐ}_{k}},\delta_{𝒮}\right)$, and (ii) samples from the out-of-sample predictive distribution ${\tilde{y}}_{i}^{-{ℐ}_{k}}\sim p_{ℳ}\left({\tilde{y}}_{i}|y^{-{ℐ}_{k}}\right)$. Because of the simplifications afforded by (4)-(5) and (11), computing ${\overset{ˆ}{\delta }}_{𝒮}^{-{ℐ}_{k}}$ only requires the out-of-sample expectations ${\hat{y}}_{i}^{-{ℐ}_{k}}≔E_{\overset{˜}{y}|y^{-{ℐ}_{k}}}\left\{\overset{˜}{y}\left({\overset{˜}{x}}_{i}\right)\right\}$ or ${\hat{h}}_{i}^{-{ℐ}_{k}}≔E_{\overset{˜}{y}|y^{-{ℐ}_{k}}}h\left({\tilde{y}}_{i}\right)$ for the classification setting. For many models ℳ, there is a further simplification that $E_{\overset{˜}{y}|y^{-{ℐ}_{k}}}\left\{\overset{˜}{y}\left({\overset{˜}{x}}_{i}\right)\right\}=E_{\theta |y^{-{ℐ}_{k}}}\left[E_{\overset{˜}{y}|\theta }\left\{\overset{˜}{y}\left({\overset{˜}{x}}_{i}\right)\right\}\right]$, where often $E_{\overset{˜}{y}|\theta }\left\{\overset{˜}{y}\left({\overset{˜}{x}}_{i}\right)\right\}$ has an explicit form (such as in regression models). Absent such simplifications, the expectations can be computed by averaging the draws of ${\tilde{y}}_{i}^{-{ℐ}_{k}}\sim p_{ℳ}\left({\tilde{y}}_{i}|y^{-{ℐ}_{k}}\right)$.

Although these terms can be computed by repeatedly re-fitting the Bayesian model ℳ for each training/validation split k=1,…,K, such an approach is computationally demanding. Instead, we apply a sampling-importance resampling (SIR) algorithm to approximate these out-of-sample quantities. Notably, the SIR algorithm requires only a single fit of model ℳ to the complete data—which is already needed for posterior inference—and therefore contributes minimally to the computational cost of the aggregate analysis.

The details are provided in Algorithm 2. By construction, the samples ${\left\{{\tilde{y}}_{i}^{\tilde{s}}\right\}}_{\tilde{s}={\tilde{s}}_{1}}^{\tilde{S}}$ are from the out-of-sample predictive distribution $p_{ℳ}\left({\tilde{y}}_{i}|y^{-{ℐ}_{k}}\right)$. Based on Algorithm 2, it is straightforward to compute draws of ${\tilde{D}}_{{𝒮}_{1},{𝒮}_{2}}^{out}$ for any ${𝒮}_{1},{𝒮}_{2}\in S$, as well as the best subset ${\hat{\delta }}_{{𝒮}_{min}}$ in (18). Therefore, the acceptable family $A_{\eta ,\varepsilon }$ is readily computable for any η,ε. By default, we use $\tilde{S}=\left\lfloor S/2\right\rfloor$ SIR samples.

Algorithm 2 recycles computations: steps 1–2(d) are shared among all subsets 𝒮 and loss functions L. Hence, the algorithm is efficient even when the number of candidate subsets |S| is large—and notably does not require re-fitting the Bayesian model ℳ. Modifications for classification (Section 2.3) are straightforward: steps (c), (d), and (e) are replaced by $\left(c\prime \right)\;{\overset{˜}{y}}_{i}^{\tilde{s}}\sim p_{ℳ}\left({\tilde{y}}_{i}|\theta^{\tilde{s}}\right)$ for $\tilde{s}={\tilde{s}}_{1},\ldots ,{\tilde{s}}_{\tilde{S}}$ and $i=1,\ldots ,n;\;\left(d\prime \right)\;{\hat{h}}_{j}^{-{ℐ}_{k}}\approx {\tilde{S}}^{-1}\sum_{\tilde{s}={\tilde{s}}_{1}}^{\tilde{S}}h\left({\overset{˜}{y}}_{j}^{\tilde{s}}\right)$ for $j\notin {ℐ}_{k}$; and (e′) compute ${\overset{ˆ}{\delta }}_{𝒮}^{-{ℐ}_{k}}$ by solving (11) using the training data covariates ${\left\{x_{i}\right\}}_{i\notin {ℐ}_{k}}$, the weights ${\left\{\omega \left(x_{i}\right)\right\}}_{i\notin {ℐ}_{k}}$, and the pseudo-data ${\left\{{\hat{h}}_{j}^{-{ℐ}_{k}}\right\}}_{j\notin {ℐ}_{k}}$.

## Appendix B. Simulation study for classification

The synthetic data-generating process for classification mimics that for prediction: the only difference is that the data are generated as $y_{i}\overset{\text{indep}}{\sim }\text{Bernoulli}\left(\pi_{i}^{\text{*}}\right)$ with $\pi_{i}^{\text{*}}≔{\left\{1+\text{exp}\left(-y_{i}^{\text{*}}\right)\right\}}^{-1}$ and $y_{i}^{\text{*}}≔x_{i}^{\prime }\beta^{\text{*}}$ as before. For the Bayesian model ℳ, we use a logistic regression model with horseshoe priors and estimated using rstanarm (Goodrich et al., 2018). The competing estimators are constructed similarly as before, now using cross-entropy loss (10) for the proposed approach and the logistic likelihood for the adaptive lasso.

The classification performance is evaluated using cross-entropy loss for $\pi_{i}^{\text{*}}$ in Figure B.1 (top), using the same competing search methods as in Figure 1. As in the regression case, the proposed bbound(bayes) search procedure returns vastly more subsets in the acceptable family, yet maintains excellent classification performance within this collection. In addition, classification based on ${𝒮}_{min}$ and ${𝒮}_{\text{small}}$ substantially outperform the adaptive lasso. We also include the 90% interval comparisons in Figure B.1 (bottom), which confirm the results from the prediction scenario: ${𝒮}_{\text{small}}$ and Zhao et al. (2021) (modified for the logistic case) achieve the nominal coverage with the narrowest intervals.

## Appendix C. Simulation study for sensitivity analysis

We revisit the comparisons in Figure 1 to consider sensitivities to ε and ${\left\{{\overset{˜}{x}}_{i}\right\}}_{i=1}^{\tilde{n}}$. In Figure C.1, we report results for the acceptable families with ε=0.01 and ε=0.20; the frequentist estimators and post.mean are unchanged from Figure 1. Compared also to the analogous case in Figure 1 with ε=0.10, we see the expected ordering in the cardinalities of the acceptable families: larger values of ε produce a more stringent criterion and therefore fewer acceptable subsets. Notably, the main results are not sensitive to the choice of ε∈{0.01,0.10,0.20}, and ${𝒮}_{\text{small}}$ performs exceptionally well in all cases.

Next, we again modify the comparisons in Figure 1 to evaluate prediction at a *new* collection of covariates ${\left\{{\overset{˜}{x}}_{i}\right\}}_{i=1}^{\tilde{n}}$. We allow variations in the covariate data-generating process—either correlated standard normals from Section 3 or iid Uniform(0,1)—and whether the observed covariates ${\left\{x_{i}\right\}}_{i=1}^{n}$ follow the same data-generating process as the target covariates ${\left\{{\overset{˜}{x}}_{i}\right\}}_{i=1}^{\tilde{n}}$. These configurations generate four scenarios, all with ${\left\{{\overset{˜}{x}}_{i}\right\}}_{i=1}^{\tilde{n}}\neq {\left\{x_{i}\right\}}_{i=1}^{n}$. The competing acceptable families are each generated using this choice of ${\left\{{\overset{˜}{x}}_{i}\right\}}_{i=1}^{\tilde{n}}$ and the point predictions are evaluated for $y^{\text{*}}\left({\overset{˜}{x}}_{i}\right)={\overset{˜}{x}}_{i}^{\prime }\beta^{\text{*}}$ for $i=1,\ldots ,\tilde{n}=1000$. The results are in Figure C.2. Naturally, performance worsens across the board when observed and target covariates differ in either their values $\left({\left\{x_{i}\right\}}_{i=1}^{n}\neq {\left\{{\overset{˜}{x}}_{i}\right\}}_{i=1}^{\tilde{n}}\right)$ or their distributions. When the target covariates are iid uniform (top right, bottom left), all methods actually perform better—likely due to the light-tailed (bounded) and independent covariate values. Yet perhaps most remarkably, the relative performance among the methods remains consistent, while ${𝒮}_{\text{small}}$ outperforms all competitors in all but one scenario—and decisively outperforms the frequentist methods in all cases.

## Appendix D. Subset selection for classifying at-risk students

We now focus the analysis on at-risk students via the functional $h\left({\tilde{y}}_{i}\right)=I\left\{{\tilde{y}}_{i}\geq \tau_{0.1}\right\}$ under cross-entropy loss, where $\tau_{0.1}$ is the 0.1-quantile of the reading scores. The posterior predictive variables ${\tilde{y}}_{i}$ derive from the same model ℳ as in Section 4, which eliminates the need for additional model specification, fitting, and diagnostics. However, it is also possible to fit a separate logistic regression model to the empirical functionals $h\left(y_{i}\right)$, which we do for the logistic adaptive lasso competitor.

It is most informative to study how the acceptable families $A_{0,0.1}$ differ for targeted classification relative to prediction. The comparative distributions of ${\tilde{D}}_{{𝒮}_{min},𝒮}^{out}$ (Figures 4 and D.1) clearly show that classification admits smaller subsets capable of matching the performance of ${𝒮}_{min}$ within 1%.

The variable importance metrics (Figures D.2 and E.3) identify the same keystone covariates as in the prediction setting. However, although there are many more acceptable subsets for classification $\left(\left|A_{0,0.1}\right|=1547\right)$ than for prediction $\left(\left|A_{0,0.1}\right|=1183\right)$, each ${\text{VI}}_{\text{incl }}\left(j\right)$ is generally much smaller for classification. Indeed, the smallest acceptable subset for classification is smaller, $\left|{𝒮}_{\text{small}}\right|=16$, and is accompanied by 21 acceptable subsets of this size. In aggregate, these results suggest that near-optimal linear classification of at-risk students is achievable for a broader variety of smaller subsets of covariates.

Lastly, the selected covariates from ${𝒮}_{\text{small}}$ are plotted with 90% intervals in Figure D.3. Compared to the prediction version, ${𝒮}_{\text{small}}$ for classification replaces 1st trimester ${\text{PM}}_{2.5}$ exposure with acute ${\text{PM}}_{2.5}$ exposure and omits the interactions Hisp × Blood_lead and Hisp × EconDisadv, but is otherwise the same. The posterior expectations under ℳ are excluded because they are not targeted for prediction of $h\left({\tilde{y}}_{i}\right)$ and therefore are not directly comparable. However, there is some disagreement between ${𝒮}_{\text{small}}$ and the adaptive lasso, the latter of which excludes several keystone covariates and again suffers from inconsistency between the point and interval estimates.

## Appendix E. Supporting figures

These figures include: pairwise correlations among the covariate (Figure E.1) and co-variable importance for prediction (Figure E.2) and classification (Figure E.3).
